# An overview of recent advances in duplex DNA recognition by small molecules

**DOI:** 10.3762/bjoc.14.93

**Published:** 2018-05-16

**Authors:** Sayantan Bhaduri, Nihar Ranjan, Dev P Arya

**Affiliations:** 1 NUBAD, LLC, 900B West Faris Rd., Greenville 29605, SC, USA; 2 National Institute of Pharmaceutical Education and Research (NIPER), Raebareli 122003, India; 3 Clemson University, Hunter Laboratory, Clemson 29634, SC, USA

**Keywords:** alkylators, antibiotic, anticancer, antineoplastic, antiproliferative, DNA recognition, groove binders, hairpin polyamides, Hoechst 33258, intercalators

## Abstract

As the carrier of genetic information, the DNA double helix interacts with many natural ligands during the cell cycle, and is amenable to such intervention in diseases such as cancer biogenesis. Proteins bind DNA in a site-specific manner, not only distinguishing between the geometry of the major and minor grooves, but also by making close contacts with individual bases within the local helix architecture. Over the last four decades, much research has been reported on the development of small non-natural ligands as therapeutics to either block, or in some cases, mimic a DNA–protein interaction of interest. This review presents the latest findings in the pursuit of novel synthetic DNA binders. This article provides recent coverage of major strategies (such as groove recognition, intercalation and cross-linking) adopted in the duplex DNA recognition by small molecules, with an emphasis on major works of the past few years.

## Review

### 1. Introduction

DNA is one of the central components of cellular machinery and storage unit of genetic information. It plays key roles in replication, transcription, protein-coding and cell integrity as well as in carrying the genetic blueprint for inheritance. The DNA–protein interactions involve high fidelity protein readout of the base edges exposed in the major and minor grooves of the DNA. Such interactions are also augmented by a series of electrostatic and van der Waals interactions including salt bridge formation with the phosphate backbone [1]. Although, the majority of proteins recognize DNA in the major groove due, in large part, to the potential and shape complementarity, several others also recognize the minor groove by sufficiently distorting the DNA structures leading to the opening of the minor groove [2]. In addition to the conventional direct and indirect readout mechanism, proteins have also been proposed to recognize the DNA minor groove by sensing variations in the shape and electrostatics [3].

The coding regions of the human genomic DNA contain highly conserved sequences that express proteins, which are essential for the cell survival and maintenance. Over or under expression of proteins has been linked to several disease states including cancer [4]. Therefore, control of gene expression has been long perceived and successfully demonstrated as a means of therapeutic development. Since DNA–protein interactions involve significant contacts in the major and minor grooves of DNA for error-free readout, small molecules (natural and synthetic) that bind strongly in the grooves have been discovered and designed to competitively inhibit such interactions. Additionally, molecules that are capable of insertion between the DNA base pairs can also disfavor DNA–protein interactions directly or allosterically. Consequently, small molecule DNA binders have been in the limelight of drug-discovery programs due to their ability to act as gene expression inhibitors [5].

The recognition of DNA is both shape and sequence dependent as DNA polymorphism leads to significant changes in the groove structure. DNA is broadly categorized to possess three major forms: A, B and Z which differ from one another in several ways such as helical sense, pitch, groove width, base orientation and sugar pucker (Table 1). The major differences in the two generally encountered A- and B-forms of DNA is in the sugar pucker and their groove widths. In A-form DNA, the major groove is narrower but has a wide/shallow minor groove. In contrast, the minor groove of B-DNA is narrow and becomes even narrower in DNAs with contiguous AT stretches (termed as the B* form of the DNA) where the width of the narrow groove reduces to approximately 2.8 Å from a usually observed width of approximately 5.7 Å [6]. In contrast to the A- and B-form DNA, Z-DNA is a left handed structure formed by alternating G and C base pairs and contains some features of both A- and B-DNA such as the sugar pucker and a slightly bigger number of base pairs per turn [7].

**Table 1:** A table showing the differences in the A-, B- and Z-form DNA [7–8].

	A-form	B-form	Z-form

helix sense	right-handed	right-handed	left-handed
base pairs/turn	11	10.4	12
pitch per turn of Helix	25.3 Å	35.4 Å	45.6 Å
glycosyl bond	*anti*	*anti*	alternating *anti* and *syn*
sugar pucker	C3'-*endo*	C2'-*endo*	C:C2'-*endo*, G:C3'-e*ndo*
major groove	narrow and very deep	wide and quite deep	flat
minor groove	very broad and shallow	narrow and quite deep	very narrow and deep

**Figure 1:**
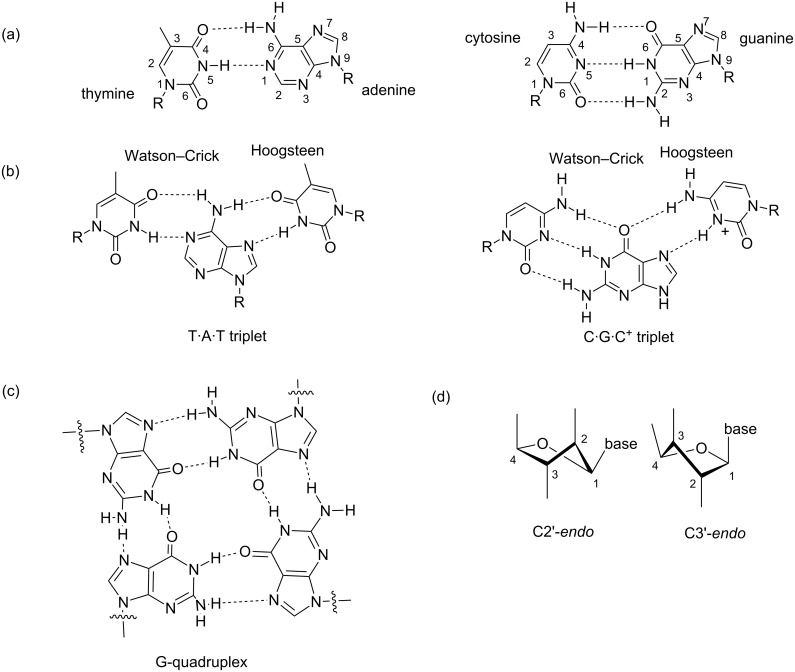
A figure showing the hydrogen bonding patterns observed in (a) duplex (b) triplex and (c) quadruplex DNA structures. (d) Conformations of sugar pucker in DNA.

The discovery of multistranded DNA structures such as G-quadruplexes [9], which uses eight Hoogsteen-paired hydrogen bonds to form a tetrad (Figure 1) has further enhanced our understanding of the diversity of DNA shapes and structures. In a parallel tetramolecular quadruplex d(TG_4_T), the features of nucleotides at each base resemble that of the B-DNA (C2’-*endo* sugar pucker, *anti* orientation and ≈12 Å groove width). However, in quadruplex fold-back structures, unusual loop connectivity gives rise to extremely wide grooves in addition to narrow and medium grooves [10] in which the width of wide grooves goes up to approximately 18 Å, far exceeding the groove widths found in B-DNA structures. These variations in the groove widths and shapes shed light on the challenges in programmed DNA recognition in a sequence and shape selective manner.

DNA recognition by small molecules can be divided in two broad categories: covalent and non-covalent. Covalent binding (e.g., cis-platin binding to guanine bases) to DNA is irreversible and causes permanent stall of transcription leading to cell death. Non-covalent interaction between small molecules and DNA is usually reversible and can further be classified as minor groove binders, intercalators, backbone binders, and major groove binders. There are reports of natural and designed molecules that display multivalency in DNA recognition by binding at more than recognition sites (minor groove, major groove or base pair insertion) [11–13]. In synthetic multivalent ligands, which are made to enhance DNA affinity, tether length and composition play a significant role in target selectivity and specificity.

Several focused reviews on small molecule DNA binding agents have been published in recent years. A few have updated the progresses made in disease specific DNA binders [14–15] while others have included class specific or site-specific DNA binding agents [16–23]. A few others have covered nucleic acids binders in general [20] as well as an emerging therapeutic DNA target: the DNA G-quadruplex [24]. In this review, we provide a detailed overview of discoveries made in the search of duplex DNA recognition agents (groove binders, intercalators and alkylating agents), which includes both classical DNA binders and new advancements in the recent years (with emphasis on research advances reported in the last five years). For a focused work, we have excluded triplex and quadruplex DNA binders for this review. In particular, we cover the advances made in DNA minor groove recognition using new analogues and derivatives of classical minor groove binders such as distamycin, netropsin, polyamides, bisbenzimidazoles and organic cations. We have also included new intercalating agents as well as major groove binding ligands especially the multivalent ligands that can simultaneously recognize one or more sites on DNA leading to strong affinity for DNA. We finally shed light on new reports of DNA alkylating agents towards the end of this review. While it is impossible to absorb the vast expanse and comprehensiveness of reports on all DNA binding agents, this review article intends to provide a substantial coverage of new advancements made in the discovery of major leads in three most visited areas (groove recognition, intercalation and cross linking agents) of DNA recognition.

### 2. Minor groove binders (MGBs)

**Figure 2:**
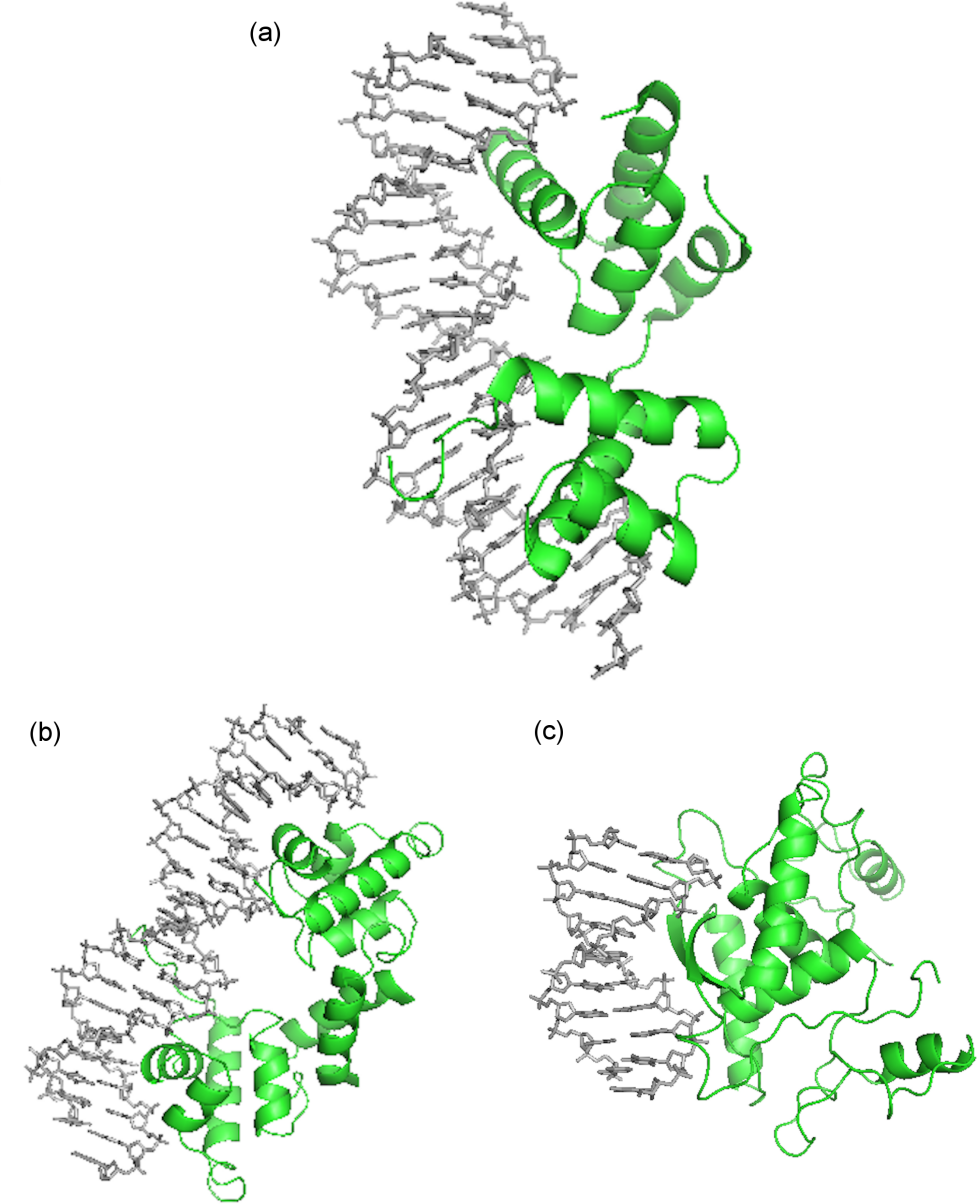
(a) Portions of MATα1–MATα2 are shown contacting the minor groove of the DNA substrate. Key arginine residues within this region facilitate the interaction (PDB ID# 1AKH) [25]; (b) Figure shows DNA bound to λ repressor protein. Alpha helices of the protein dimers recognize specific sequences within the DNA major groove (PDB ID# 1LMB) [26]; (c) The MetJ dimer β sheet contacts the DNA ligand major groove via side chains on the face of the β sheet (PDB ID# 1CMA) [27].

DNA groove binding small molecules comprise various heterocyclic and/or aromatic hydrocarbon rings with limited rotational freedom and torsion, allowing these drugs to fit into major/minor grooves of DNA by displacing water molecules from the spine of hydration as shown in the Figure 2 [25–27]. These molecules bind to the edges of the base pairs of the DNA duplex (usually G·C sites in the major groove, A·T sites in the minor groove) via reversible non-covalent interactions. These binding interactions reduce the conformational freedom of the small molecules and usually are opposed by an unfavorable entropic cost. However, these energetic costs are balanced and outweighed by favorable contributions from the hydrophobic transfer of drugs from solution to DNA-binding site [28–29]. Groove binding usually does not influence huge structural/conformational changes in the DNA duplex; this mode of binding may be considered similar to a standard lock and key recognition [30].

Minor groove binding drugs (MGBs) are usually isohelical, crescent-shaped molecules, which are compatible with the shape of the minor groove. Binding of MGBs and proteins occurs primarily via H-bonds, electrostatics, van der Waals and hydrophobic interactions (Figure 2). Figure 2a shows that the arginine side chain of the MATα2 N-terminal arm facilitates interaction between portions of the heterodimer MATα1–MATα2 with the minor groove of the DNA substrate by forming alternate H-bond interactions [25]. The main characteristic feature of MGBs is their preference for narrow A·T-rich regions compared to G·C regions because (i) they can form hydrogen bonds to N3 of adenine and O2 of thymine in the A·T region; (ii) less steric hindrance in the A·T region in comparison to the G·C region due to the presence of an extra protruding C2-amino group of the guanine base [19].

#### 2.1. Polypyrroles and polyamides

**Figure 3:**
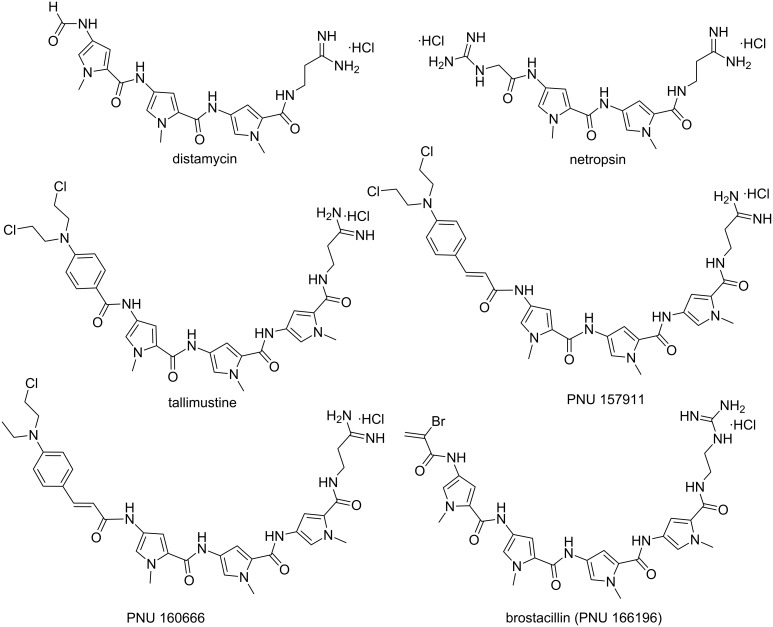
Chemical structures of naturally occurring and synthetic hybrid minor groove binders.

The first two MGBs discovered were distamycin A and netropsin (Figure 3). These naturally occurring molecules are characterized by repeating *N*-methylpyrrole units with one or more positively charged nitrogen atoms at the end. Their concave-shaped aromatic framework fits perfectly in the convex-shaped minor groove of double-stranded DNA. Therefore, these drugs have been referred to as “shape-selective” binders [31]. They selectively interact with A·T-rich regions containing at least four A·T base pairs in the minor groove via hydrogen bonding interaction between the groove floor base pairs and the amides and electrostatic stabilizing interactions between the protonated amines under physiological pH and negatively charged phosphate backbone as reported by NMR and crystallographic studies [32–36]. These molecules were shown as inhibitors of Werner and Bloom syndrome helicases and dual topoisomerase I/II inhibitors [37–38].

In order to improve DNA binding affinity and sequence specificity with reduced side effects, a series of synthetic hybrid molecules derived from distamycin and netropsin was synthesized and their biological activities were thoroughly studied both in vitro and in vivo. One significant representative of this class is tallimustine (FCE 24517, TAM), which is a benzoyl nitrogen mustard derivative of distamycin characterized by an oligopeptidic pyrrolocarbamoyl framework ending with an amidino moiety [39–40]. The benzoyl nitrogen mustard (BAM) to the formyl end of the distamycin acts as an alkylating moiety whereas the distamycin framework acts as a DNA binding domain. Therefore, due to the installation of the alkylating moiety, TAM has higher cytotoxic activity in comparison to distamycin, and shows a broad spectrum of in vitro and in vivo antitumor activities. Tallimustine retains the preference for A·T-rich regions in the minor groove that alkylates N3 of adenine in a highly sequence specific manner, thereby inhibiting the binding of transcription factors such as OTF-1 and NFE1 on specific AT-rich sequences [41–42]. However, clinical development of TAM was discontinued due to severe myelotoxicity.

With distamycin, netropsin and TAM as the lead compounds for novel anticancer drugs, a plethora of oligopyrrole derivatives were reported with the aim of increasing stability, greater DNA binding affinity, sequence specificity, more cytotoxicity and minimizing the unwanted physiological side effects [43]. It has been observed that drugs with high degree of sequence specific binding affinity and selective alkylation of DNA could inhibit the binding of the regulatory proteins to DNA. Several researchers have investigated the effect of adding alkylating groups [44] such as traditional nitrogen mustards [45] to α-halogenoacrylic [46] moieties by keeping the distamycin and netropsin frameworks intact. Cinnamic mustard (PNU 157911) and half-mustard (PNU 160666, Figure 3) derivatives of distamycin show excellent antileukemic activity and are found to be significantly less myelotoxic than TAM against murine and human hematopoietic progenitor cells [43]. The positively charged basic amidino side chain, responsible for electrostatic interaction with negatively charged DNA phosphate backbone, was also replaced by various amidine-like groups, such as cyanoamidine, *N*-methylamidine, *N*,*N*-dimethylamidine, and guanidino moieties either to increase the stability, cytotoxicity and enhance solubility at physiological pH. Comparable cytotoxicity was observed in these cases suggesting a general behavior of these classes of molecules including the amidine modification. In addition, a novel class of cytotoxic MGBs comprising of α-bromo or chloroacrylamide moieties linked to distamycin were identified. Among all different synthetic analogs, brostacillin (PNU-166196, Figure 3) was found to be a potent anticancer drug due to its improved cytotoxicity/myelotoxicity ratio [47–48]. Brostacillin acts as an effective DNA alkylator only in presence of high levels of cellular thiols such as glutathione [49]. Moreover, it was thirty-fold more active in comparison to TAM in inducing apoptosis in A2780 human ovarian carcinoma cells [43]. Khalaf et al. reported a new class of neutral, non-cationic minor groove binders derived from distamycin where the cationic tail group has been replaced by a neutral, polar variant including cyanoguanidine, nitroalkene, and trifluoroacetamide groups. These conjugates exhibit significant antibacterial activity against Gram-positive bacterial strains [50].

**Figure 4:**
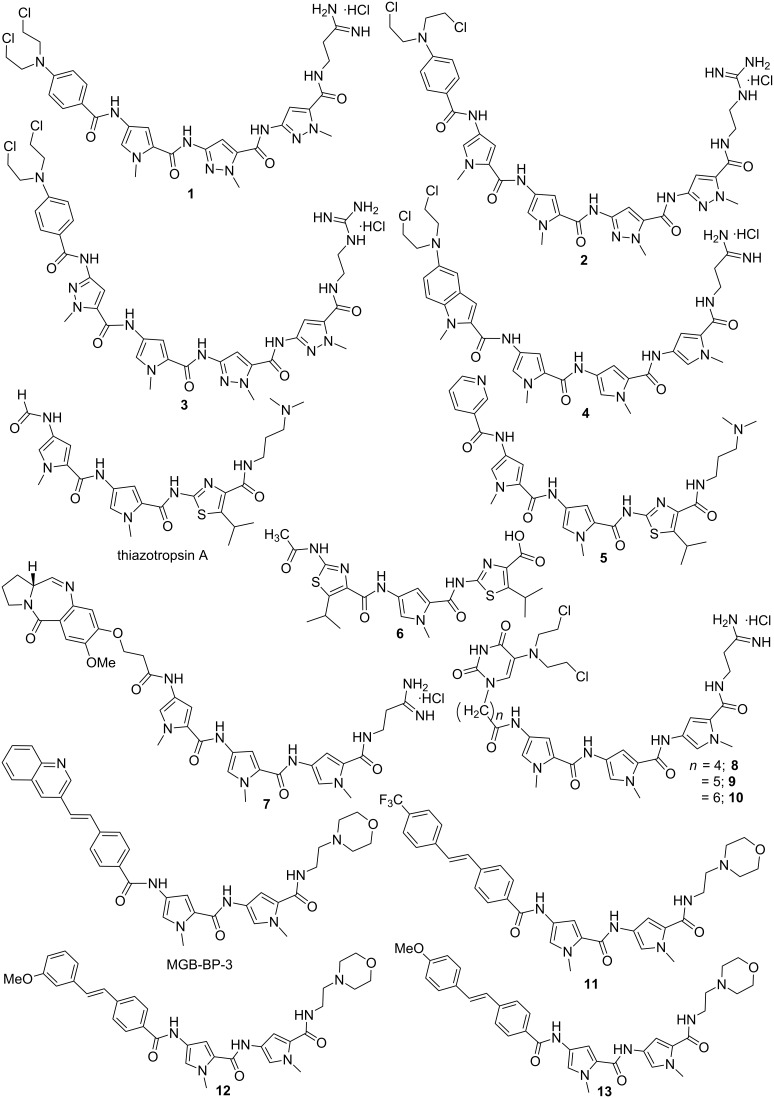
Synthetic structural analogs of distamycin A by replacing one or more pyrrole rings with other heterocycles or by tethering with known antitumor agents.

Several other distamycin analogs were synthesized by replacing one or more pyrrole rings with other heterocycles such as pyrazoles [51], benzofurans [52], thiazoles, thiophenes, imidazole and oxazoles [53] in order to establish a structure–activity relationship. It has been observed that the number and position of pyrrole rings are crucial for antileukemic activity. The presence of pyrrole rings close to the alkylating BAM moiety is responsible for better cytotoxic activity both in vitro and in vivo, whereas a pyrazole ring in close proximity to BAM drastically reduces the same as shown in the Figure 4 (**2** > **1** > **3**) [51]. Baraldi et al. designed and synthesized a series of novel compounds comprising different benzoheterocyclic rings, bearing a nitrogen mustard, a benzoyl nitrogen mustard or an α-bromoacryloyl group as alkylating moieties, tethered to a distamycin framework. Conjugate **4** (a 5-nitrogen mustard *N*-methylindole derivative) was found to exhibit excellent antileukemic activity with a very long survival time in comparison to tallimustine [52]. Khalaf et al. reported several heterocyclic trimeric distamycin analogs with enhanced lipophilicity [53]. These structural analogs comprise of branched *N*-alkyl- and *N*-cycloalkylpyrroles to test the conformational flexibility towards DNA binding. Hydrophobic N-terminal amides and substituted thiazole replacing pyrrole were installed in order to impart more lipophilicity.

All these compounds were shown to bind A·T-rich regions preferentially. The compounds containing branched *N*-alkylpyrrole, hydrophobic N-terminal amide, and especially *C*-isopropylthiazole (thiazotropsin A as shown in the Figure 4) showed significant antimicrobial activity against MRSA and *Candida albicans* strains*.* Thiazotropsin A has shown much higher affinity than parent distamycin A (preferential selectivity towards G·C sites) due to the presence of an isopropyl-substituted thiazole ring, which makes the molecule more hydrophobic [54]. Recently, a small set of analogs of thiazotropsin was designed and synthesized to study their solution-phase self-association characteristics and DNA molecular-recognition properties [17]. The authors showed a measurable difference in solution-phase self-assembly character with enhanced DNA association characteristics by replacing the formamide head group in thiazotropsin A with nicotinamide as shown in the Figure 4 (conjugate **5**). Suckling et al. further demonstrated another structural analog of thiazotropsin conjugate **6**, a heterocylic triamide containing thiazole carboxylic acid, which showed significant activity (MIC = 63 nM) against *Trypanosoma brucei* [55]. However, the authors reported other conjugates with two thiazoles directly linked via an amide bond, which retained activity to a lesser extent. Baraldi et al. designed and synthesized a novel conjugate **7** by combining naturally occurring antitumor agent distamycin A with the pyrrolo[2,1-*c*][1,4]benzodiazepine moiety (PBD), related to the naturally occurring anthramycin for investigating its antitumor activity [56]. Conjugate **7** demonstrated much better activity compared to distamycin in vitro by inhibiting cell growth of neoplastic cell lines and preferentially binding to G·C-rich sequences in the minor groove. In similar fashion, they further reported a series of novel hybrids by tethering distamycin A with the antineoplastic agent uramustine via a flexible polymethylene chain of variable length (*n* = 1 to 6) in order to test their DNA binding affinity and cytotoxicity [57].

It has been observed that hybrid conjugates **8**, **9** and **10** with longer linkers exhibit relatively higher cytotoxicity in comparison to both distamycin and uramustine. The distamycin fragment directs binding to the A·T-rich sequences in the minor groove, and higher flexibility due to the longer linker allows optimal positioning of the mustard for DNA alkylation. In addition, longer linker imparts more lipophilicity, which in turn, favors better transportation of these compounds into the cells. Anthony et al. reported a series of short MGBs based on the lead compounds distamycin and thiazotropsins with the installation of hydrophobic aromatic head groups, including quinolyl and benzoyl derivatives, and alkenes as linkers in order to investigate their antimicrobial properties [58–59]. One of these structural analogs, MGB-BP-3 (Figure 4), containing a stilbene like fragment as head group and two *N*-methylpyrroles attached to an aminoethylmorpholine as tail group, was found to be extremely potent (MIC values in the range of 0.5–13 μg mL^−1^) against several strains of *S. aureus*, both methicillin-sensitive and resistant strains. High antimicrobial activity, shown by this drug, was due to the presence of a hydrophobic head group with a hydrogen-bonding substituent (3-quinolinyl nitrogen forming a hydrogen bond with a guanine amino group at the base of the minor groove) and a low p*K*_a_ tail group. This drug was further selected for the treatment of Gram-positive bacteria *Clostridium difficile* infections and is currently in the phase II clinical trials. Szerszenowicz et al. developed a new set of potential minor groove binders derived from netropsin and bis-netropsin analogs by replacing *N*-methylpyrrole rings with other heterocyclic rings and their antiproliferative activity was tested on MCF-7 breast cancer cells [60]. Suckling et al. recently designed and synthesized a series of structurally diverse MGBs, derived from distamycin, in order to test their lung cancer inhibition activity against the melanoma cancer cell line B16-F10 [14]. Conjugate **11** was found to be extremely potent and exhibits 70-fold activity in comparison to the standard therapy, gemcitabine. Thus, the conjugate **11** was chosen for further development as an anti-lung cancer therapeutic. In the similar fashion, the same group investigated the correlation between DNA binding and antibacterial activity shown by these novel distamycin alkene-containing analogs (MGB-BP-3, **12** and **13,** Figure 4). This has been attributed to strong self-association (dimerization) in an antiparallel, head-to-tail orientation in aqueous solution during complex formation with duplex DNA oligomers verified via NOE experiments [61]. They further reported several structurally diverse MGBs, derived from distamycin, in order to probe their antifungal and antimycobacterial activity; several of these novel conjugates showed promising activity against the fungus *C. neoformans* (MIC_80_s ranging from 0.25–4 μg/mL) and the mycobacterium *M. tuberculosis* (MIC_99_s 3.1 μM) [62].

**Figure 5:**
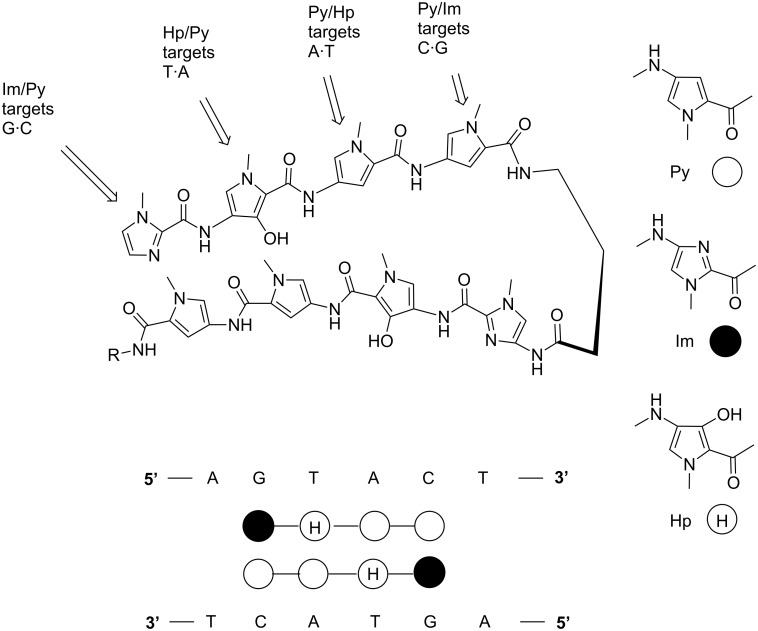
Pictorial representation of the binding model of pyrrole–imidazole (Py/Im) polyamides based on the base pairing recognition rule in the minor groove.

Since the last few decades, a plethora of synthetic structural analogs of distamycin, netropsin and thiazotropsins were developed to test their DNA binding affinity, sequence specificity and cytotoxicity, thereby eventually developing a general approach for the regulation of gene expression by DNA binding small molecules. However, all these analogs do not possess the ideal crescent shape required to wrap around the minor groove of DNA, which limit their efficacy to recognize longer stretches of DNA sequence. In order to achieve better sequence specificity, a series of oligomeric “hairpin (HP)” polyamides containing pyrrole and imidazole ring systems (Py/Im) were designed and synthesized by Dervan et al. and followed by other groups. It was observed that pyrrole/imidazole polyamides were able to bind side-by-side in the minor groove of DNA with high affinity and in a sequence-specific manner. Crystal structure studies confirmed the existence of a hydrogen bond between the Im nitrogen and the exocyclic amine of guanine. Dervan et al. have further developed rules for base pairing recognition of minor groove binding polyamides where antiparallel side-by-side pairings of pyrrole (py) and imidazole (Im) amino acids successfully distinguish G·C from C·G base pairs, and both of these from A·T/T·A base pairs as depicted in Figure 5 [63]. Again, a Py/Py pair specifies A·T from G·C but does not distinguish A·T from T·A. Thus, in order to break this degeneracy, Dervan et al. successfully introduced another aromatic amino acid, 3-hydroxypyrrole (Hp). With this subtle change by replacing a single hydrogen atom with a hydroxy group, hydroxypyrrole–imidazole–pyrrole polyamides form four ring pairings (Im/Py, Py/Im, Hp/Py and Py/Hp) and are able to distinguish all four Watson–Crick base pairs in the minor groove of DNA [64–66]. These polyamides are a successful class of synthetic DNA (minor groove) binders that can be designed to bind chosen DNA sequences via directed H-bonds, shape complementarity, and can compete with specific protein–DNA binding interactions in the minor or major grooves [67–68].

**Figure 6:**
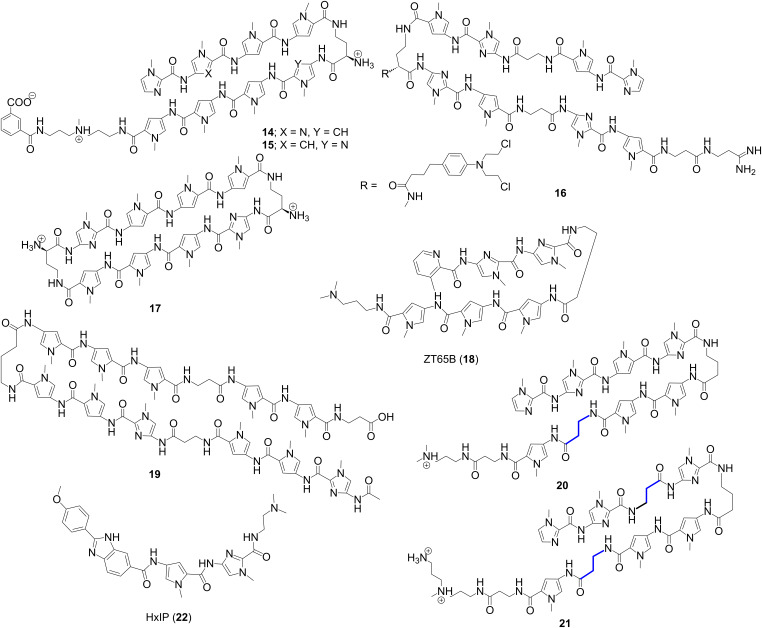
Chemical structures of synthetic “hairpin” pyrrole–imidazole (Py/Im) conjugates.

**Figure 7:**
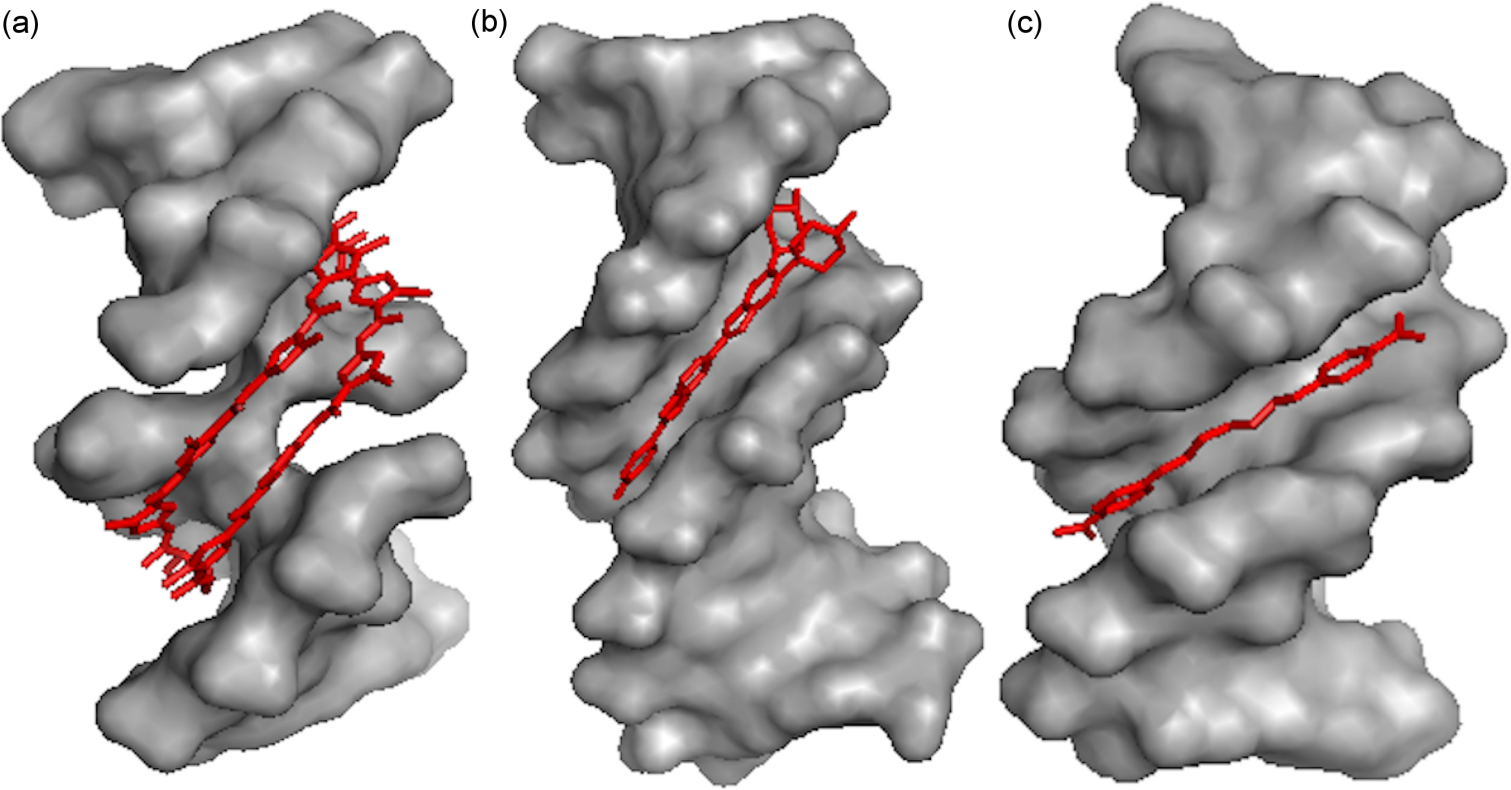
(a) Minor groove complex formation between DNA duplex and 8-ring cyclic Py/Im polyamide (conjugate **17,** PDB ID# 3I5L) [76]; (b) Complex between DNA duplex and Hoechst 33258 (PDB ID# 8BNA) [82]; (c) Crystal complex between DNA duplex and pentamidine (PDB ID# 3EY0) [83].

A variety of sequence-specific Py/Im polyamides were designed and synthesized in order to interfere with transcription factor binding and to regulate gene expression, both in vitro and in vivo. These polyamides are shown to bind DNA with comparable and/or even higher affinities than those of natural DNA-binding transcription factors. Dickinson et al. designed novel polyamides, which were able to bind adjacent to the recognition sites of a broad-range of transcription factors TBP, Ets-1, LEF-1 and NF-κB [69], thereby inhibiting binding of these transcription factors to DNA and ternary complex formation [70]. Dervan et al. has further introduced a novel Py/Im polyamide **14** (Figure 6) that was able to bind preferentially the sequences 5′-WGGWWW-3′ and 5′ GGGWWW-3′ in the Nuclear factor κB sites, thereby reducing the expression of various NF-κB-driven genes including IL6 and IL8 [71]. Another structural analog of conjugate **11**, conjugate **15** was developed to interrogate its effect on the activity of RNA polymerase II [72]. Lenzmeier et al. provided strong evidence for inhibition of Tax protein–DNA minor groove interaction via synthetic Py/Im polyamides, which is believed to be essential for treating and/or preventing HTLV-I-associated diseases [73]. Gottesfeld et al. synthesized a series of Py/Im HP-polyamide–DNA alkylator (chlorambucil) (HP-Chl) conjugates in order to bind and alkylate within the HIV-1 promoter region, thereby blocking HIV-1 replication and screened them against human colon carcinoma cell lines [74–75]. It has been observed that conjugate **16** showed significant changes in cellular morphology and causes cells to arrest in the G2/M stage of the cell cycle. The authors further confirmed via microarray analysis that the histone H4c gene is significantly downregulated by the conjugate **16** which was assumed to be bound to and alkylate a site in the H4c promoter in treated cells, thereby inhibiting tumor growth in mice. Chenoweth and Dervan showed DNA structural distortion induced by an 8-ring cyclic Py/Im polyamide (conjugate **17**) bound to the central 6 bp of the sequence d(5*'*-CCAGGCCTGG-3*'*)_2_ by using a high resolution X-ray crystal structure as shown in Figure 7a [76]. This allosteric perturbation of the DNA helix by small molecules through binding at distinct locations on promoter DNA provides a clear understanding of how transcription factor activity could be disrupted and gene expressions could also be regulated. In order to target the inverted CCAAT box (ICB) of the human multidrug resistance 1 gene (MDR1) promoter and to distinguish between different promoter ICB sites, several ICB-containing DNA hairpin polyamides were designed with different flanking base pairs. It was confirmed via thermal-denaturation studies and DNase I-footprinting assays that one of these conjugates containing a 3-methylpicolinate moiety (ZT65B, compound **18**) binds in the minor groove and effectively targeted ICBa and ICBb, similar to the 3*'*-ICB site of MDR1 (TGGCT) [77].

Lai et al. synthesized the novel hairpin Py/Im polyamide conjugate **19** and a mismatch conjugate in order to target -545 to -539 base pairs of human transforming growth factor-beta1 (hTGF-beta1) promoter and diminish the gene and protein expression [78]. The authors went on to confirm that conjugate **19** binds its corresponding target sequence whereas the mismatch conjugate fails to recognize the sequence by using a gel mobility shift assay. Additionally, conjugate **19** drastically inhibited the promoter activity of hTGF-beta1 as well as gene and protein expression as determined via in vitro transcription experiments and luciferase assay. This research paved the way for a novel gene therapy for the treatment of TGF-beta-related diseases. Several researchers have installed a flexible β-alanine fragment on Py/Im HP polyamides for better recognition of DNA by reducing molecular rigidity. However, in order to get a better understanding of how β-substitution diversely affects the HP–DNA binding affinity, selectivity, and especially kinetics, Wilson and co-workers conducted a thorough study by synthesizing eight heterocyclic HP polyamides having single and double β-substituted derivatives with their cognate and mutant sequences [79]; two of the representative conjugates **20** and **21** are shown in the Figure 6. In conclusion, the authors reported that β-substituted polyamides weakens the binding affinity of these conjugates with cognate DNA and drastically influence the binding kinetics such as association and dissociation rates in a position- and number-dependent manner. The authors, in addition, replaced the monocationic Dp group [3-(dimethylamino)propylamine] in conjugate **20** with a dicationic Ta group (3,3*'*-diamino-*N*-methyldipropylamine) in conjugate **21** to minimize the frequently observed polyamide aggregation. This subtle modification retains the polyamide-DNA binding mode and affinity by reducing aggregation and also helps to conduct a detailed thermodynamic study for the 8-ring HP polyamides for the very first time. Recently, Hartley et al. designed and synthesized a hybrid fluorescent HP polyamide conjugate **22** (Figure 6) by attaching the A·T recognizing fluorophore, *p*-anisylbenzimidazolecarboxamido (Hx) in order to target the inverted CCAAT box 2 (ICB2) of the topoisomerase IIα (topo IIα) promoter and to monitor the cellar uptake of the conjugate [80–81]. Gratifyingly, conjugate **22** targets the 5'-TACGAT-3' sequence of the 5' flank of ICB2 with high affinity and sequence specificity, thereby disrupting the NF-Y-ICB2 interaction. In addition, cellular uptake and nuclear localization of conjugate **22** could be easily monitored as a result of its inherent fluorescence property.

Despite myriad important biological roles of hairpin and cyclic Py/Im polyamides in regulating natural gene expression via sequence-specific DNA binding, the lack of viable strategies for facile synthesis of library of structural variants of these classes of conjugates remains a huge challenge for the researchers. In order to resolve this issue, Dervan et al. recently published a modular microwave-assisted Fmoc-based solid phase synthetic approach for the syntheses of cyclic Py/Im polyamides [84]. This group previously optimized and reported a machine-assisted Fmoc solid phase synthesis of simpler polyamides to afford high step-wise coupling yield [85]. A seven-member library of cyclic polyamides targeting androgen response element (ARE) and the estrogen response element (ERE) was synthesized in 12–17% overall yield. Selective modifications could also be done on the GABA turn units, which showed improved cellular uptake properties.

**Figure 8:**
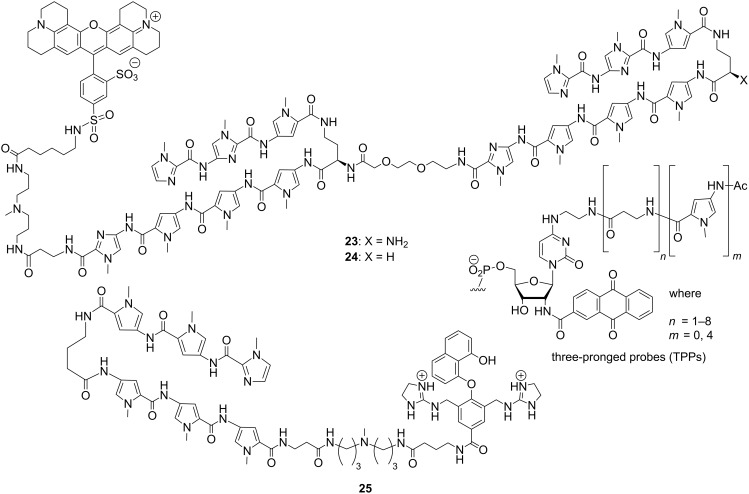
Telomere-targeting tandem hairpin Py/Im polyamides **23** and **24** capable of recognizing >10 base pairs; **25**: a novel DNA-cleaving agent comprising of bis(guanidinium)alcohol tethered with Dervan hairpin polyamide; three-pronged probes (TPPs) to target A·T-rich sequences selectively.

Sugiyama et al. designed and synthesized a series of telomere-targeting synthetically challenging tandem hairpin Py/Im polyamides which could recognize >10 base pairs with flexible linker conjugated with a fluorescent dye (either Texas Red (TR) or Cyanine 3 (Cy3)) using a Fmoc-based solid phase synthetic approach; two of the representative conjugates **23** and **24** are shown in the Figure 8 [86–87]. The authors investigated the binding affinity and sequence specificity of these conjugates for the human telomeric repeat TTAGGG in mouse MC12 and human HeLa cells. In mouse and human cells, TR-conjugated polyamides **23** and **24** successfully targeted to the corresponding telomeres and highlighted the telomere foci clearly because of their fluorescent nature. Later on, the authors successfully designed tandem tetramer Py–Im polyamides with 4 hairpins and 3 hinges targeting 24 bp of the human telomere sequences [88]. Thus, the authors set the new record for the longest binding site of synthetic, non-nucleic-acid-based, sequence-specific DNA-binding molecules. These conjugates could bind to four telomeric repeats with nanomolar dissociation constants, confirmed via SPR analysis. In the similar fashion, Nozeret et al. reported a series of nine fluorescent hairpin polyamides by attaching cyanine and fluorescein dyes to target mouse major satellite DNA using thermal denaturation, gel-shift electrophoresis, circular dichroism and fluorescence spectroscopy [89–90]. Some of these fluorescent probes were found to detect target sequences in mouse living cell lines and the nuclear substructures formed by repeated DNA sequences in living cells were nicely visualized. Choice of fluorophores attached to the N-terminus of the polyamides remains extremely crucial, as they seem to affect DNA minor groove binding significantly. In order to design a novel DNA cleaving agent, a bis(guanidinium)alcohol tethered with Dervan hairpin polyamide was synthesized. The resulting conjugate **25** binds A·T-rich DNA duplexes with comparable affinity to that of the parent polyamide and breaks one strand of double-stranded plasmid DNA by interacting with anionic phosphodiesters in a fast transphosphorylation step as contact ion pairs at micromolar to high nanomolar concentration range [91]. Richert et al. designed a novel set of three-pronged probes (TPPs) comprising of cap, β-alanines and oligopyrrolamides in order to bind A·T-rich target strands from three sides (Watson–Crick face, terminus, and minor groove) resulting in exceptionally stable duplexes (Δ*T*_m_ = +44.8 °C) and high selectivity [92].

**Figure 9:**
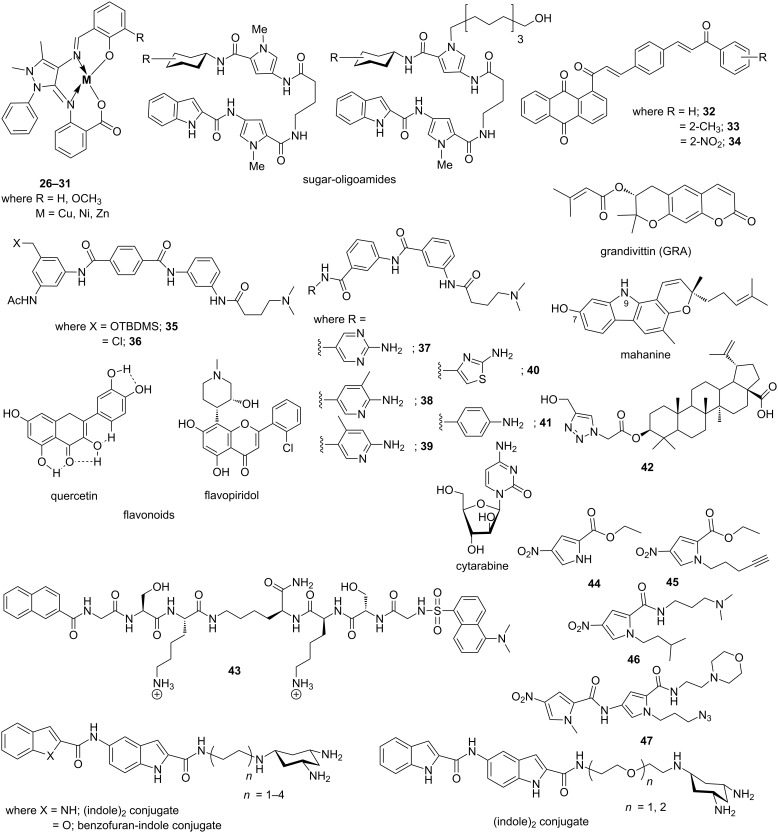
Representative examples of recently developed DNA minor groove binders.

Six novel 4-aminoantipyrine derived Schiff bases and their metal complexes with Cu(II), Ni(II), Zn(II) ions (conjugates **26**–**31**) were synthesized and characterized and binding of these complexes with ct-DNA were analyzed by electronic absorption spectroscopy, viscosity measurement, cyclic voltammetry and molecular modeling (Figure 9) [93]. Docking results confirmed that these complexes have the ability to interact with the minor groove of the ct-DNA. In addition, the authors confirmed that in presence of ascorbic acid, these complexes could facilitate DNA cleavage. Moreover, these complexes showed improved biocidal activity than the free ligands against various bacterial strains such as *Staphylococcus aureus*, *Pseudomonas aeruginosa*, *Escherichia coli*, *Staphylococcus epidermidis*, and *Klebsiella pneumonia*. Nair et al. synthesized and characterized three mononuclear copper(II) complexes, [Cu(tpy)Cl_2_], [Cu(tpy)(NO_3_)_2_(H_2_O)] and [Cu(Ptpy)Cl_2_]·H_2_O·HCl and investigated their cytotoxicity and primary mode of DNA binding mechanism [94]. Molecular modeling as well as DNA cleavage studies have revealed that the first two complexes are DNA minor groove binders, whereas the third complex prefers an intercalative mode of binding to DNA. All these complexes show nuclease activity in the presence of hydrogen peroxide and induce apoptosis to human A549 lung adenocarcinoma cells. A series of novel glyco-oligoamides (Figure 9) has been designed and synthesized in order to investigate the molecular basis of carbohydrate–minor groove DNA interactions by Vicent et al. [95].

NMR spectroscopy and molecular modeling studies further confirmed the existence of directional intramolecular hydrogen bonds and CH–π interactions, which results in stabilizing these conjugates in the minor groove by marinating a stable hairpin structure [96]. The authors tethered various monosaccharides such as β-xylose, α-xylose, β-galactose, β-glucose and β-L-fucose to a minor groove binding residue, Py-γ-Py-Ind, structurally analogous to distamycin and netropsin. A new set of novel anthraquinone–chalcone hybrids were synthesized using Claisen–Schmidt reaction in order to test their anticancer potential against human cancer cell lines and DNA binding affinity and specificity. It has been observed that three conjugates **32**–**34** exhibited significant cytotoxicity against LS174 and HeLa cancer cell lines by interacting non-covalently with the minor groove of the double helical ct-DNA [97]. Barker et al. have designed a series of novel di- and triaryl benzamide MGBs differing in the polar side chain, bonding and substitution patterns and functionalization of benzylic substituents and evaluated their antiproliferative activity as well as their DNA binding affinity [98]. It has been confirmed that the most active conjugates are unsymmetrical triaryl benzamides **35** and **36** comprising of a bulky and alkylating chlorobenzylic substituent, respectively, and a polar amino side chain. Conjugate **35** with a bulky OTBDMS benzylic substituent was found to be the most active agent with (IC_50_ 5.0 μM) followed by conjugate **36** with a chloro substituent (IC_50_ 9.9 μM). Drozdowska et al. reported a series of distamycin analogues **37**–**41** (Figure 9) as potential minor groove binders and their minor groove DNA binding affinity as well as antiproliferative effects on human MCF-7 breast cancer cells were evaluated [99]. These conjugates bind within the minor groove of B-DNA. They inhibited catalytic action of endonucleases in A·A, A·T, T·T and A·G restriction sites but failed to block G·C-rich sequences. In addition, they act as potent topoisomerase II inhibitor at the concentration 10 μM and show antiproliferative and cytotoxic activities in breast cancer cell line in the range of 81.70 μM and 200.00 μM. Conjugate **41** with a 6-aminophenyl moiety appeared to be the most effective among others. Suckling et al. designed a set of 31 Strathclyde minor groove binders (S-MGBs), derived from distamycin, by varying the head groups (amidine, amide, or alkene), heterocyclic building blocks and their alkyl substituents and the basicity of the C-terminal tail group in order to investigate their antimalarial activity against a chloroquine sensitive (3D7) and resistant (Dd2) strain of *Plasmodium falciparum* [100]. Conjugates with an alkene link between the two N-terminal building blocks and a *C*-alkylthiazole moiety appeared to the most active among others with IC_50_ values in the range of 30–500 nM. The same group further demonstrated that the head group plays a crucial role in determining the activity against *Trypanosoma brucei* with another set of novel S-MGBs, structurally analogous to distamycin [101]. Coumarins are a group of phenolic compounds with excellent cytotoxic and antiviral properties. Again, dihydrofuranocoumarins are another class of coumarins possessing anticancer activities. Recently, Ahmadi et al. identified several dihydrofuranocoumarins, especially grandivittin (GRA), from *Ferulago macrocarpa* (Fenzl) Boiss., and their mechanism of minor groove DNA binding and antibacterial, cytotoxic and antioxidant activities were evaluated [102]. A molecular docking study has revealed that GRA interacts with ct-DNAs via hydrogen bonding interactions between the oxygen atoms of GRA and adenine bases of DNA and van der Waals interactions. Moreover, GRA significantly reduces the polymerization activity of DNA polymerase as a result of binding to minor groove DNA. Samanta et al. investigated a thorough structure–activity correlation between mahanine, an anticancer carbazole alkaloid, and its chemically modified analogs to test the role of various functional groups on its antiproliferative activity against 19 cancer cell lines [103]. It has been shown that the C-7 hydroxy and the 9-NH group showed significant contribution towards its DNA minor groove binding ability via strong association with the phosphate backbone. In addition, the presence of these functional groups could enhance antiproliferative activity of cancer cells towards apoptosis through the mitochondrial pathway. Mitrasinovic has reported sequence-dependent binding of various structurally different flavonoids (quercetin (QUE) and flavopiridol (FLP)), a family of prospective anticancer agents, to duplex DNAs [104]. The five hydroxy groups in QUE involve in the intramolecular hydrogen bonding which is attributed to its planar orientation whereas the chlorophenyl moiety, the heterocyclic fragment with the C5 and C7 hydroxy groups and C8 piperidinyl substituent in FLP favor non-planar binding geometry. The author examined their sequence-specific binding affinity using sophisticated molecular dynamics approach with eight different nucleotides having variety of sequences. It has been observed that QUE appears to be a minor groove binder, whereas FLP involves in combined mode of interaction such as minor groove binding and intercalation. A set of betulinic acid analogs were synthesized by using azide–alkyne click reaction and their anticancer activities against different cancer cell lines and normal human PBMC cell line were evaluated by MTT assay. Conjugate **42** was found to be extremely potent against HT-29 cell line with an IC_50_ value of 14.9 μM and its cytotoxicity was attributed to DNA minor groove binding ability [105]. Recently, Schmuck et al. have developed a first prototype of cationic oligopeptide-based molecular beacon (conjugate **43**) coupled with a FRET pair, a naphthalene donor and a dansyl acceptor, for ratiometric detection of ds-DNA by fluorescence microscopy with preference for A·T-rich sequences [106]. Two positively charged lysine residues are expected to interact with ds-DNA electrostatically. Upon binding to the minor groove of ds-DNA, the conformation of conjugate **43** was changed from an extended to a folded form, thereby changing the efficiency of the FRET process between the two fluorophores and exhibiting a significant red shift in the emission spectrum. Moreover, the conjugate **43** could be used as an attractive tool for imaging of nuclear DNA in the cells due to its low cytotoxicity. A series of water-soluble peptidocalix[4]arenes with arginine-rich short narrow groove binding residues on the lower rim of the calix[4]arene scaffold were reported by Soltani et al. in order to study the binding between well-matched and mismatched DNA duplexes [107]. Fluorescent titrations, ethidium bromide (EB) displacement assays, DNA-melting experiments, and circular dichroism (CD) analysis revealed these conjugates are high affinity sequence specific DNA groove binders and could successfully recognize a C·C mismatch in a DNA duplex. Recently, the binding mechanism of the anticancer drug cytarabine with calf thymus DNA (ct-DNA) was investigated in vitro by Shahabadi et al. by multispectroscopic techniques and molecular modeling study [108]. It has been shown that cytarabine acts in a groove-binding mode, which was confirmed by fluorescence experimental results of Hoechst 33258 displacement by the drug. Hydrophobic interactions play a crucial role in its binding to DNA groove. Similarly, the same group recently reported a macrocyclic copper(II) complex, ([CuL(ClO_4_)_2_] where L is 1,3,6,10,12,15-hexaazatricyclo[13.3.1.1^6,10^]eicosane) and studied its interaction with calf thymus DNA (ct-DNA). It was confirmed that the Cu(II) complex could displace the ct-DNA-bound Hoechst33258 suggesting it binds to the minor groove of ct-DNA via groove binding mechanism [109]. Suckling et al. have recently reported four nitro­pyrrole-based compounds (conjugates **44**–**47**, Figure 9) as building blocks for the synthesis of novel minor groove binders [110]. Crystal structure data revealed that nitro groups and ester moieties in conjugates **44** and **45** are coplanar with the pyrrole ring, whereas the isopropyl fragment in conjugate **46** lies out of the pyrrole plane. Coplanarity extends to the second pyrrole ring in case of conjugate **47** and all these conjugates form layer-like structures during crystal formation via multiple hydrogen bonding interactions. This structural information indeed helps to design novel MGBs with much better binding affinity and specificity. A new family of conjugates between a Zn(II)-tach complex and (indole)_2_ or benzofuran–indole amide minor groove binders connected through alkyl or ethoxyethyl linkers were developed by Tecilla et al. [111]. The authors confirmed that these conjugates with tach units, either free or Zn(II)-complexed forms, bind strongly to the minor groove through electrostatic interactions with the phosphate backbone and the binding affinity strongly depends upon the nature and length of the linkers.

#### 2.2. Bisbenzimidazoles

**Figure 10:**
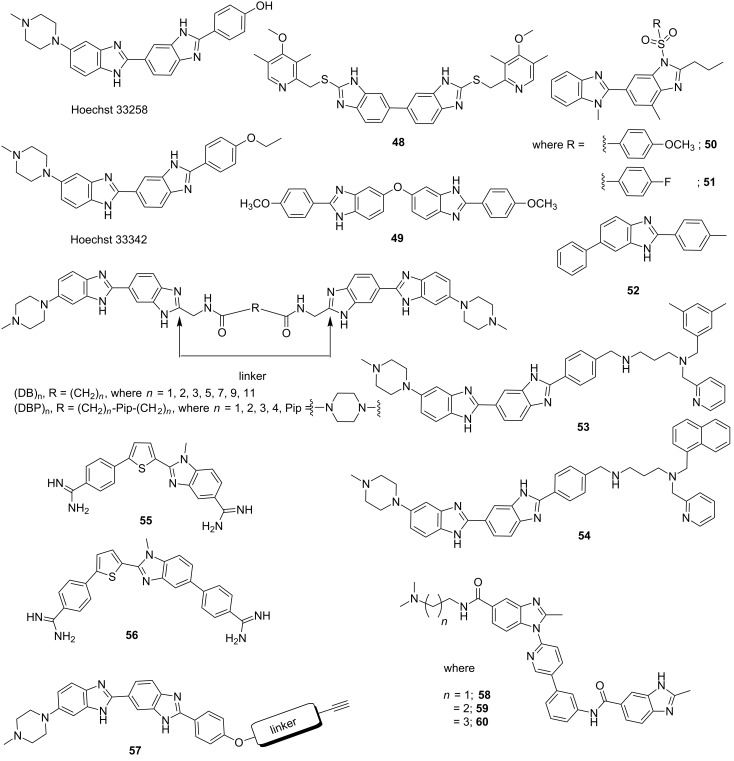
Chemical structures of bisbenzamidazoles Hoechst 33258 and 33342 and their synthetic structural analogs by varying substitutions and linkers.

Bisbenzimidazoles are one of most extensively studied DNA minor groove binding compounds; Hoechst 33258 and 33342 are representatives of this class of compounds as shown in Figure 10. Minor groove complex formation between DNA duplex and Hoechst 33258 is shown in Figure 7b [82]. X-ray crystallographic and NMR studies confirmed that Hoechst 33258 binds to the A·T-rich sequences in minor groove with the planar benzimidazole groups are oriented parallel to the direction of the groove. Hoechst 33258 primarily acts as human topoisomerase I poison [112] and initially showed cytotoxicity against L1210 murine leukemia; however, after passing human phase I clinical trials for pancreatic cancer, it failed to produce any effective result in phase II trials [113]. However, due to its high binding affinity to B-DNA duplexes, several groups have designed various structural analogs of Hoechst 33258 in order to achieve a better sequence-specific DNA binder with reduced toxicity [114]. Yang et al. reported a series of novel symmetrical bisbenzimidazoles as DNA minor groove binders. A molecular modeling study confirmed that conjugate **48** could dock into the minor groove of DNA. These conjugates exhibited cytotoxic activities on SKOV-3, HeLa, and BGC-823 cell lines in vitro in the single-digit micromolar range [115]. Another set of bisbenzimidazoles was synthesized by varying substitutions on the phenyl ring where the two benzimidazoles were linked via an oxygen atom. Most of these conjugates showed significant antitumor activity in vitro compared to Hoechst 33258. Amongst them, conjugate **49** (Figure 10) was found to be most potent with IC_50_ values of 0.56 μM for HL60 (Human promyelocytic leukemia cells) tumor cell line and 0.58 μM for U937 (Human leukemic monocyte lymphoma cells) tumor cell line with reduced toxicity in comparison to paclitaxel and 5-FU [116]. Ivanov et al. reported two different sets of strong minor groove binders, derived from well-known DNA minor groove binder Hoechst 33258. These conjugates are fluorescent dimeric bisbenzimidazoles [(DB)_n_ and (DBP)_n_] tethered by oligomethylene linkers of varied lengths with or without a central 1,4-piperazine residue [117]. The low solubility of (DB)_n_ in aqueous solution due to aggregation has forced the authors to introduce a 1,4-piperazine residue in the oligomethylene linkers (DBP)_n_, making them tetracations instead of dications for (DB)_n_ at neutral pH. By the virtue of their higher solubility in aqueous media, (DBP)_n_ could easily penetrate cell and nuclear membranes of living cells and inhibit in vitro eukaryotic DNA topoisomerase I and prokaryotic DNA methyltransferase (MTase) at micromolar concentrations. Rangappa et al. recently reported the synthesis of a series of novel bisbenzimidazole derivatives and evaluated their antiproliferative and antiangiogenic activity properties. Conjugates **50** and **51** were found to be not only potent antiproliferative agent against HeLa, HCT116 and A549 cells, but also did not exhibit cytotoxicity towards non-diseased (Vero) cells [118]. In addition, the authors tested the efficacy of these two lead conjugates **50** and **51** against Ehrlich ascites tumor (EAT) bearing mice for its antitumor and antiangiogenic properties and concluded that these conjugates drastically reduced the cell viability, body weight, ascites volume and downregulated the formation of neovasculature and production of Vascular Endothelial Growth Factor (VEGF).

They further reported another novel benzimidazole derivative conjugate **52** which could inhibit topoisomerase II activity and in vitro transcription by binding to the DNA minor groove [119]. Conjugate **52** could successfully exhibit cytotoxicity in leukemic cells by inducing apoptosis. Amirbekyan et al. reported a novel groove binding anchoring strategy for DNA-based asymmetric catalysis by synthesizing various structural analogs of Hoechst 33258. It has been observed that amine analogs (conjugate **53** and **54**) showed higher affinity towards ct-DNA and poly[d(A·T)_2_] in comparison to alkyne analogs with reduced flexibility and one less charged nitrogen atom, thereby reducing strength of electrostatic interactions between the ligands with DNA phosphate backbone [120]. Wilson et al. rationally designed benzimidazole derivatives by keeping pre-organized *N*-methylbenzimidazole (*N*-MeBI)-thiophene as central fragment (conjugates **55** and **56,** Figure 10) in order to selectively bind mixed G·C and A·T sequences of DNA. They hypothesized that thiophene (positive electrostatic potential) and the electron-donor nitrogen of N-MeBI should pre-organize the conformation for accepting hydrogen bond from G-NH_2_, which was validated by replacing the thiophene moiety with other heterocycles, resulting in lowering the binding affinity and specificity [121]. Arya et al. reported a series of Hoechst 33258 based mono- and bisbenzimidazole derivatives and their *E. coli* DNA topoisomerase I inhibition, binding to B-DNA duplex, and antibacterial activity has been evaluated [122]. It has been observed that the conjugates with alkynyl side chains show excellent *E. coli* DNA topoisomerase I inhibition properties with IC_50_ values of <5.0 μM, which was attributed to critical interactions between the inhibitor side chain and amino acids of the active site of DNA topoisomerase I, as suggested by the modeling study. In general, bisbenzimidazole derivatives (conjugate **57**) exhibit much better antibacterial activity than mono-benzimidazoles for Gram-positive strains. More importantly, the linker lengths and composition have dramatic influence on DNA binding and cell uptake, suggesting that the roles of the linkers should be carefully investigated when combining fragments in drug discovery applications [123]. Recently, Picconi et al. reported a series of nontoxic triaryl benzimidazole conjugates derived from existing classes of MGBs, to probe their antibacterial activity against multidrug resistant (MDR) Gram-positive and Gram-negative species; conjugates **58**–**60** (Figure 10) showed excellent antibacterial activity with MICs ranging from 0.5–4 μg/mL for Gram-positive strains and MICs ranging from 16–32 μg/mL for Gram-negative strains [124]. However, molecular modeling revealed that these conjugates could not bind into the minor groove due to change in their conformation, thereby showing negligible DNA binding. Thus, their antibacterial activity is not attributed to DNA binding affinity due to lack of DNA stabilization by these conjugates.

#### 2.3. Bisamidines

**Figure 11:**
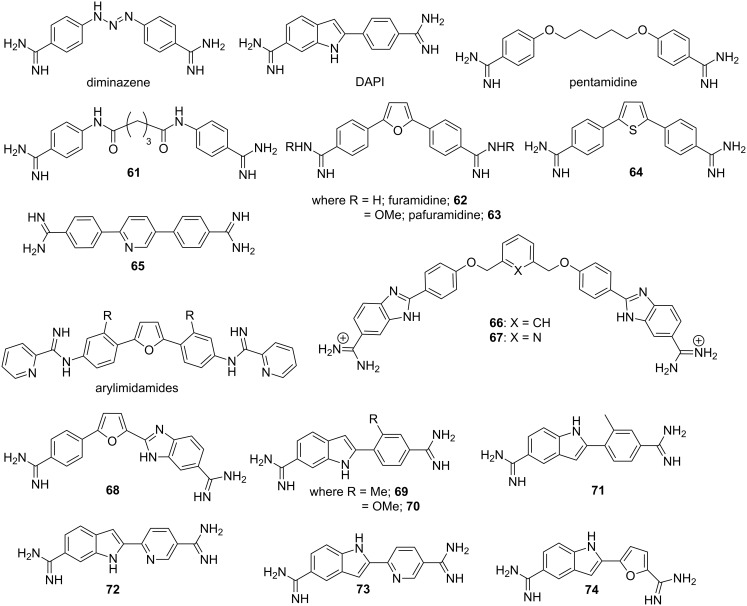
Chemical structures of bisamidines such as diminazene, DAPI, pentamidine and their synthetic structural analogs by varying substitutions, linkers and introducing heterocycles.

One of the oldest known clinically relevant small molecule MGBs with immense biological applications is the aryl bisamidine class related to diminazene, DAPI and pentamidine as shown in Figure 11. Minor groove complex formation between DNA duplex and pentamidine is shown in Figure 7c [83]. These small molecules are known to bind A·T-rich sequences preferentially. Moreno et al. reported a coiled-coil structure formed by the complex of the DNA duplex with pentamidine. The authors showed that the central part of the pentamidine binds to the minor groove, whereas the charged terminal amidine groups interact electrostatically with negatively charged phosphates, thereby stabilizing the complex through the formation of cross-links between neighboring duplexes [83]. However, due to intrinsic toxicity, various structural analogs of pentamidine were designed over the years by replacing the ether linkage with bis-amide **61** [125], introducing heterocyclic rings such as furan **62** and **63** [126], thiophene **64** [127] and pyridine **65** (Figure 11) [128].

These conjugates exhibit potent antibacterial and antiprotozoal activity with much reduced toxicity. It was further concluded that π-stacking, H-bonding with the floor of the minor groove along with appropriate curvature helps them to bind with specific DNA sequence [129]. A series of arylimidamide analogues were synthesized and their binding affinities towards DNA minor groove was studied by Wilson et al. via fluorescence displacement titration, circular dichroism, DNase I footprinting, biosensor surface plasmon resonance, X-ray crystallography and molecular modeling [130]. These experiments revealed that these novel conjugates form 1:1 complexes with A·T sequences in the DNA minor groove, and the binding strength depends upon substituent size, charge and polarity. In addition, they have also exhibited improved uptake properties in *Leishmania* and *Trypanosoma cruizi* than existing heterocyclic diamidines. With this success, this group further rationally designed several other minor groove binders in order to achieve even better specificity, which could bind to two A·T sites separated by G·C base pairs. Molecular modeling and other biophysical studies confirmed that the conjugate **67**, pyridyl analog of conjugate **66**, could successfully recognize a single G·C base pair flanked by A·T sequences via several van der Waals and hydrogen bonding interactions [131]. Wilson et al. further designed a novel dicationic diamidine (conjugate **68**) to recognize a mixed base pair site for the first time. It has been confirmed via ESIMS that the conjugate **68** binds in the minor groove of ATGA sequences as a dimer with positive cooperativity [132]. Recently, they reported a series of structural analogs of DAPI by replacing the phenyl ring with substituted phenyl and heterocyclic rings as shown in the Figure 11. Amongst them, conjugates **69**–**74** are found to bind in the minor groove with improved affinity. Additionally, these conjugates exhibit superior in vitro antitrypanosomal activity in comparison to DAPI itself [133].

**Figure 12:**
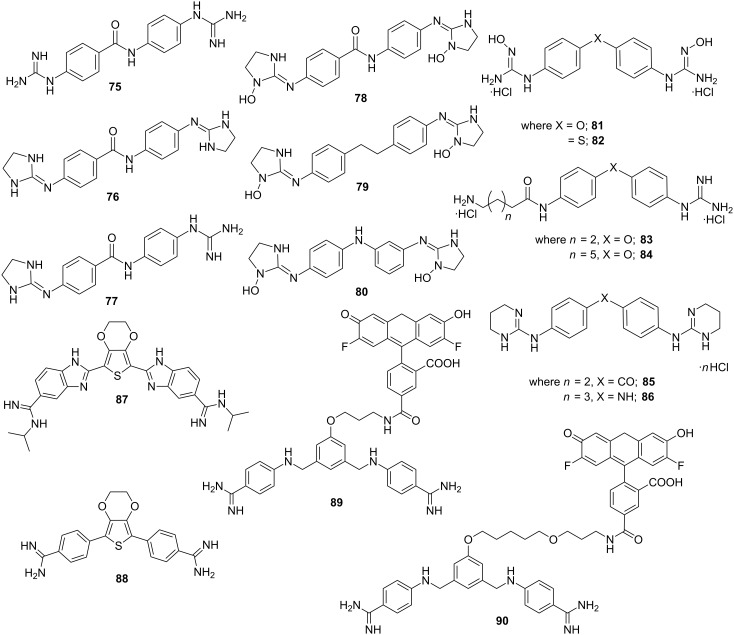
Representative examples of recently developed bisamidine derivatives.

Rozas et al. designed and synthesized a new family of asymmetric peptide-linked diaromatic dications with a linear core as potent DNA minor groove binders (Figure 12) [134]. Various biophysical experiments such as surface plasmon resonance and circular dichroism revealed that due to the presence of a planar amide linker between the phenyl rings, these newly synthesized bis-cationic ligands (conjugates **75**–**77**) showed a much improved preferential minor groove binding ability towards A·T-rich regions in comparison to other guanidinium-like derivatives with curved cores. Dardonville reported a series of high affinity DNA minor groove binders N-substituted bisimidazoline arylamides to test the effect of imidazoline ring N-substitution on preferentially binding at A·T sites over G·C sites [135]. The authors demonstrated N1 hydroxylation could enhance DNA binding affinity and selectivity towards AATT sites over (A·T)_4_ sequences (conjugates **78**–**80**). Rozas et al. further reported the syntheses of a new family of hydroxyguanidinium aromatic derivatives as potential minor groove binders and cytotoxic agents; two of the representative structures **81** and **82** are shown in the Figure 12. These conjugates showed antiproliferative effects in human promyelocytic HL-60, breast carcinoma MCF-7, and neuro-blastoma cell lines, although no direct correlation between their cytotoxicity and DNA binding affinity was established yet [136]. With the initial success, they reported DNA minor groove binding aminoalkyl derivatives of diaromatic guanidines **83** and **84**, which exhibit significant antiprotozoal activity in vitro against *P. falciparum* and *T. b. rhodesiense* strains [137]. Moreover, the authors further developed a new family of dicationic bis-2-amino-1,4,5,6-tetrahydropyrimidines with more suitable size and lipophilicity to bind in the minor groove than the previously reported conjugates [138]. Thermal denaturation experiments and DFT calculations revealed that conjugates **85** and **86** appeared to be much better binders than bis-guanidiniums, but weaker in comparison to bis-2-aminoimidazolinium derivatives as reported earlier [139]. Recently, a series of novel amidine derivatives of 3,4-ethylenedioxythiophene with excellent antibacterial activities against Gram-positive (including resistant MRSA, MRSE, VRE strains) and Gram-negative bacterial strains has been reported [140]. The bisbenzimidazole derivatives (conjugate **87**) exhibited the widest spectrum of activities whereas bis-phenyl derivatives were the most potent ones (conjugate **88**). In addition, these conjugates demonstrated excellent DNA binding ability (Δ*T*_m_ = 15.4 °C) through various electrostatic and hydrogen bonding interactions. Bordello et al. designed two fluorescence-labeled bisbenzamidine (BBA) derivatives (conjugates **89** and **90,** Figure 12) tethered with the dye Oregon Green (OG) separated via linkers of various lengths in order to develop highly sensitive sequence-specific DNA binders [141]. Detailed photophysical analysis revealed that these conjugates enforce a significant fluorescence enhancement upon binding to the minor groove of ds-DNA with excellent sequence specificity and reduced affinity constants in comparison to the parent BBA without the dye. Recent work from the Poon and Wilson groups has also shown how these designed amidines can be used to target TF activity [142].

#### 2.4. Aureolic acid group of anticancer drugs

**Figure 13:**
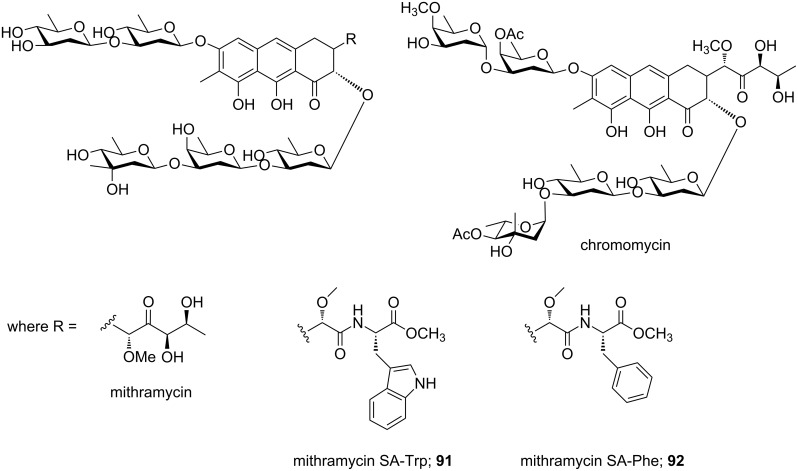
Chemical structures of chromomycin, mithramycin and their synthetic structural analogs **91** and **92**.

The antineoplastic and antibiotic natural products mithramycin (MTM) and chromomycin act as minor groove binder with the preference for G·C-rich sequences and represent aureolic acid group of anticancer drugs (Figure 13) [114]. Aich and Dasgupta established two different types of mithramycin-Mg^2+^ complex formation by which MTM exhibits its cytotoxic effect by interacting with DNA minor groove as a divalent metal coordinated dimer, thereby regulating gene expression [143].

Recently, mithramycin was identified as a selective inhibitor of abnormal oncogenic transcription factor EWS–FLI1 in Ewing sarcoma. Hou et al. designed two different mithramycin analogs **91** and **92** in order to probe the mechanism for MTM recognition of DNA to understand how MTM interferes with EWS–FLI1 [144–145]. The authors reported crystal structures of conjugates **91** and **92** bound to DNA sequence specifically and also confirmed a ternary complex formation in the minor groove between FLI1–DNA–MTM on a single GGAA FLI1/MTM binding site. This research introduces a new approach to selectively target EWS–FLI1 or other oncogenic transcription factors to develop anticancer therapeutics.

### 3. Intercalators

Another mode of non-covalent reversible interaction between DNA and small molecules is intercalation. In general, DNA intercalators consist of planar aromatic or heteroaromatic groups capable of stacking between the adjacent DNA base pairs. These complexes are stabilized by π–π stacking interactions, van der Waals forces, hydrophobic interactions and/or charge transfer forces [29,146].

**Figure 14:**
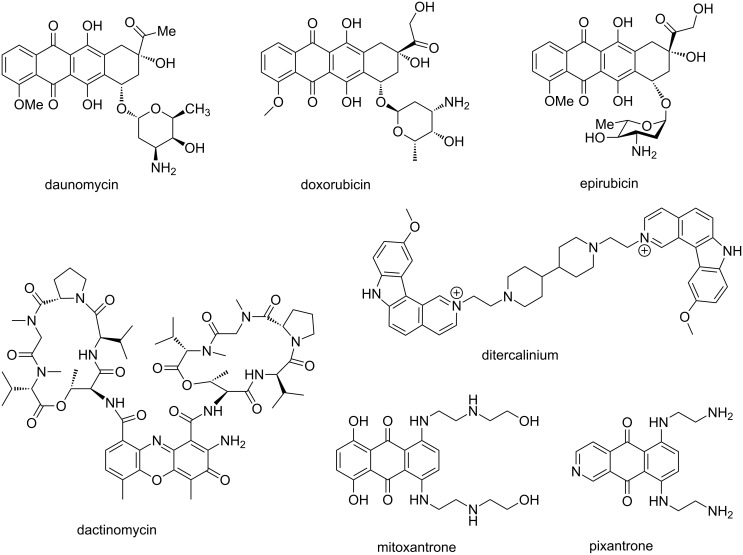
Chemical structures of well-known naturally occurring DNA binding intercalators.

DNA intercalation induces local structural perturbations in the DNA helix; mainly decrease in the helical twist, which results in lengthening of the DNA [147]. These structural modifications lead to the interruption of DNA replication, transcription and DNA repair processes by interfering with the function of DNA-associated proteins such as polymerases, transcription factors and topoisomerases [19]. Therefore, DNA intercalators are often used as chemotherapeutic agents. Several DNA intercalating drugs have been identified over the years, which include daunomycin (trade name Cerubidine), doxorubicin (trade name Adriamycin), epirubicin (anthracycline family), dactinomycin (trade name Cosmegen), ditercalinium, bleomycin, elsamicin A, m-AMSA, mitoxantrone, acridines, ethidium bromide and so on (Figure 14) [30,148–151]. Anthracyclines are a class of antitumor antibiotics, isolated from *Streptomyces* species, mostly used in various cancer chemotherapy such as acute leukemia, Hodgkin’s and non-Hodgkin’s lymphoma, breast and ovarian cancer, lung cancer, gastric (stomach) cancer, testicular cancer, bladder cancer and soft tissue sarcoma etc. In addition, they act as topoisomerase II inhibitors [152]. Daunomycin and doxorubicin both possess a planar ring, a fused cyclohexane ring system and an amino sugar moiety. The ionic interaction between the protonated amine group on the carbohydrate residue and the negatively charged DNA phosphate backbone hold these drugs within the DNA groove, thereby allowing the planar aromatic ring system to intercalate within the G·C steps of the double helix [153–154]. Epirubicin, another drug in the anthracycline family, is the 4′-epimer of doxorubicin. It has been used as a chemotherapy treatment either alone or in combination with other cytotoxic agents. Epirubicin is favored over doxorubicin due to lesser side effects such as reduced myelosuppression and cardiotoxicity. Similar to the other anthracycline drugs, it also acts via intercalating into DNA strands, which eventually inhibits DNA and RNA synthesis leading to cell death [155]. Dactinomycin, also known as actinomycin D, a member of the polypeptide family, is known to inhibit DNA transcription by blocking the chain elongation. This antibiotic has a clear preference for G·C base pairs and interacts with the 2-amino group of guanine. The pentapeptide moiety interacts with the DNA minor groove by hydrogen bonding and hydrophobic interactions, whereas the phenoxazone ring slides into the G·C base pairs for intercalating. Another antitumor drug, ditercalinium, used for treatment of cancer, is an example of non-covalent DNA-binding ligand via bis-intercalation [156]. This drug is a 7*H*-pyridocarbazole dimer, which intercalates into two G·C steps in the major groove. Moreover, the positively charged bis(ethylpiperidinium) moiety interacts with the major grove via charge interaction and induce DNA repair in eukaryotic or prokaryotic cells [157–159]. These dual binding mechanisms (intercalation and minor groove binding) help to form a steady complex between these above mentioned small molecule drugs and DNA duplex. Mitoxantrone is a tricyclic planar anthraquinone derivative with two basic side chains which acts as anticancer chemotherapeutic agent via inducing DNA damage by breaking single and double strands. It is a type II topoisomerase inhibitor [160]. With reduced cardiotoxicity and functionally similar to doxorubicin, it disrupts DNA synthesis and DNA repair via intercalating between the bases in DNA duplex [161]. It has been observed that intercalating anthraquinone chromophore in a pyrimidine (3′-5′) purine sequence remains perpendicular to the direction of inter-base hydrogen bonds, whereas positively charged N-containing basic side chains project outward from the drug [162]. It shows significant activity against acute myeloid leukemia, advanced breast cancer and non-Hodgkins lymphoma [163]. Recently, Konda et al. demonstrated a binding mechanism of another anticancer drug pixantrone to three different oligonucleotide sequences by using NMR and molecular modeling. The upfield shift of pixantrone aromatic protons observed after preferential binding to symmetric CpA dinucleotide sequences supported the intercalative mode of the binding mechanism [164].

**Figure 15:**
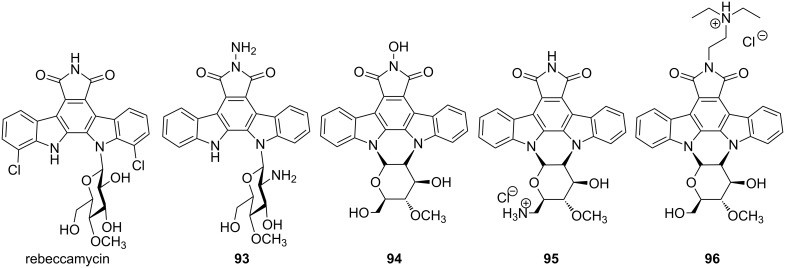
Naturally occurring indolocarbazole rebeccamycin and its synthetic analogs.

Indolocarbazoles represent a family of alkaloids containing bisindoles, which are mostly used as anticancer drugs. The natural antibiotic, rebeccamycin, isolated from *Saccharothrix aerocoloniegenes*, is a representative of this class of molecules as shown in Figure 15. This is a well-known DNA-binding agent and acts as inhibitor of topoisomerase I. The glycoside residue attached with the DNA intercalating domain plays a major role in binding of the drug to the DNA double helix, similar to daunomycin and doxorubicin. It was shown that by replacing the glucose moiety with a 2’-aminoglucose residue, DNA-binding affinity and sequence specificity of compound **93** was enhanced [165]. Another series of structural analogs were synthesized in order to develop novel tumor-active rebeccamycin derivatives. DNA binding affinity of a cationic derivative **96** containing a *N,N*-diethylaminoethyl side chain and **95** bearing an aminoglycoside moiety were compared with an uncharged analog **94**. It was observed that the cytotoxic potential of cationic **95** and **96** was higher in comparison to uncharged **94**, which is mainly attributed to the enhanced DNA binding affinity and sequence specificity. Installation of the cationic moiety on either the indolocarbazole domain or on the carbohydrate residue greatly reinforces the binding of these drugs to DNA. These molecules preferentially recognize sequences GpT·ApC and TpG·CpA steps [166].

**Figure 16:**
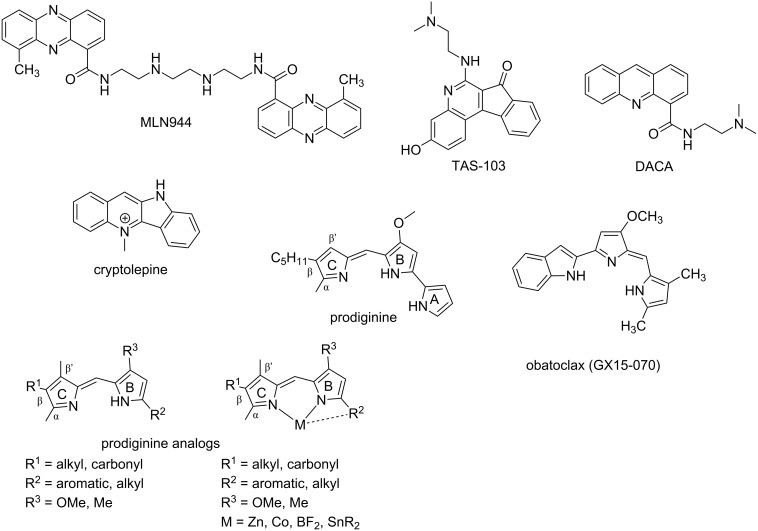
Representative examples of naturally occurring and synthetic derivatives of DNA intercalating agents.

MLN944 (XR5944) is a novel bisphenazine derivative showing excellent cytotoxic activity against various in vitro and in vivo human and murine tumor models (Figure 16) [167–168]. Sappal et al. suggested the primary mechanism of action of this drug involves DNA major groove binding via bis-intercalation and is not involved in the catalytic activity of topoisomerase I or II [169]. When applied in combination with carboplatin or doxorubicin in non-small-cell lung carcinoma [170], or in combination with 5-fluorouracil and irinotecan in colon cancer cell lines [171], MLN944 exhibited synergistic effect in vitro and in vivo. Another DNA intercalating drug TAS-103 (BMS-247615), novel quinolone derivative, is a dual inhibitor of topoisomerases I and II and shows potent cytotoxic effects in vitro and in vivo against subcutaneously-implanted murine and human tumors in vivo, as well as various lung-metastatic murine tumors [172–173]. When this drug was applied with the approved antitumor drug cis-platin, a synergistic effect was observed which could be helpful for the treatment of small-cell lung cancer.

Similar to TAS-103, DACA (*N*-[2-(dimethylamino)ethyl]acridine-4-carboxamide) is another DNA intercalating anticancer drug capable of inhibiting both topoisomerases I and II and currently in clinical trial phase II (Figure 16). It has been observed that the acridine ring intercalates between the DNA base pairs and the 4-carboxamide side chain assists in the major grove binding with its protonated *N,N*-dimethylamino group forming hydrogen-bonding interactions with guanine [174]. The prodiginine family of bacterial alkaloids, isolated from *Serratia* species, represents a varied set of heterocyclic red-pigmented natural products with potent immunosuppressive, antimicrobial and proapoptotic anticancer attributes. These 4-methoxypyrrolic natural products are structurally characterized by the presence of 4-methoxy-2,2'-bipyrrole skeleton [175]. They bind to DNA in the intercalative fashion with the preference for A·T sites. It was further confirmed that they intercalate from the minor groove, as minor groove binding drug distamycin was able to displace them from the DNA double helix. Prodiginine act as a dual topoisomerase I/II inhibitor and has been tested against more than 60 cancer cell lines including breast, lung, stomach, liver, spleen, colon, blood, and chronic myeloid leukemia with an average inhibitory concentration of 2.1 μM [176]. Obatoclax (GX15-070) is a synthetic derivative of natural prodiginines and currently under phase I and phase II clinical trials for the treatment of various types of cancer cell lines [177]. Combination therapies with other chemotherapeutic agents are also currently being tested with obatoclax. Due to their immense biological activities, numerous chemical, chemoenzymatic and biosynthetic strategies were reported to afford several structural analogs of this class of natural products [178–179]. Recently, Marchal et al. reported several structural analogs of natural prodiginines and their complexes with tin, cobalt, boron, and zinc salts with modifications at rings A and C and their antimalarial activities were evaluated in vitro using the 3D7 *Plasmodium falciparum* strain [18]. The authors went on to confirm that the presence of the nitrogen atom in the A-ring is mandatory to show antimalarial activity whereas on the contrary, the presence of an alkyl group at the β′-position of the C-ring is not essential, in fact at times detrimental. Moreover, dibutyltin complexes could also enhance the inhibitory effect in comparison to natural prodiginines, exhibiting IC_50_ values in the nanomolar range. Cryptolepine, isolated from the roots of *Cryptolepis sanguinolenta*, is an indoloquinoline alkaloid with antibacterial, antiviral, and antimalarial properties [180]. Its mode of binding to DNA was tested via absorption, fluorescence, circular and linear dichroism, as well as by a relaxation assay using DNA topoisomerases [181]. It has been observed that this alkaloid binds tightly to DNA and its primary mode of action is intercalation. Cryptolepine has a clear preference for G·C-rich sequences containing non-alternating G·C sites as demonstrated via competition dialysis assays. Besides, the positively charged nitrogen helps to maintain the stability of the DNA–ligand complex via charge interaction. Moreover, it was confirmed that this alkaloid is a potent topoisomerase II inhibitor and a promising antitumor agent [182].

**Figure 17:**
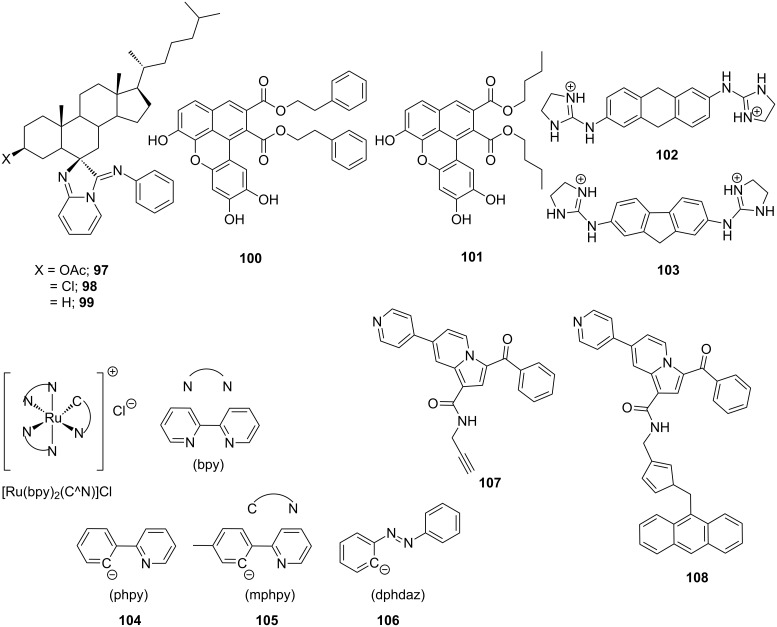
Several recent synthetic varieties of DNA intercalators.

Dar et al. designed and reported a series of novel steroidal imidazo[1',2'-*a*]pyridine derivatives (conjugates **97–99**) via an one-pot three-component tandem approach by reacting different steroidal ketones, 2-aminopyridine and isocyanides and simultaneously investigated their DNA binding affinity and in vitro cytotoxicity (Figure 17) [183]. UV–vis, fluorescence, gel electrophoresis and molecular docking studies revealed that the primary mode of binding of these conjugates with the minor groove of the DNA is intercalation, although the van der Waals and other types of electrostatic and hydrophobic interactions could also play important roles. Significant antiproliferative activity of these conjugates against different cancer cells were observed from MTT assays. These steroidal imidazopyridines induced an apoptosis in A549 cells resulting in cell cycle arrest at low concentration, respectively, confirmed via western blotting and FACS analysis. A series of novel benzo[*k*,*l*]xanthene lignans were designed and synthesized by biomimetic, Mn-mediated oxidative coupling of caffeic esters and amides by Tringali et al. and their DNA binding mechanism was thoroughly studied via DF-STD NMR analysis and molecular docking [184]. These experiments revealed their dual mode of binding mechanism; the planar core intercalates between the minor groove base pairs and the flexible protruding moieties act as minor groove binders. Moreover, conjugates **100** and **101** comprising of lipophilic esters showed significant antiproliferative activity, even better than the anticancer drug 5-fluorouracil (5-FU), against HCT-116 (colon, GI_50_ = 3.16 μM) and H226 (lung, GI_50_ = 4.33 μM) cell lines. Rozas and Wilson reported syntheses, mode of DNA binding mechanism and sequence specificity of a set of conformationally restricted symmetric and asymmetric dicationic DNA binders comprising of 9,10-dihydroanthracene (DHA) **102** and 9*H*-fluorene **103** cores; two conjugates representing each class are shown in the Figure 17 [185]. SPR studies clearly indicated the affinity of these conjugates not only for A·T oligonucleotides, but also for G·C-rich oligonucleotides. Again, they exhibited much stronger binding to DNA in comparison to the flexible core conjugates. Conjugate **103** containing a fluorene core was found to bind A·T oligonucleotides much stronger compared to DHA conjugate **102**. CD and UV experiments revealed DHA analogs bind to DNA via intercalation and fluorine analogs act as intercalator as well as minor groove binder. Nakabayashi et al. reported three cyclometalated ruthenium(II) complexes [Ru(bpy)_2_(C^N)]Cl **104**–**106** in order to study their ct-DNA binding affinity and cytotoxicity against two tumor (L1210 and HeLa) and a non-tumor (BALB/3T3 clone A31) cell lines [186]. Conjugates **104–106** primarily act as intercalators and/or minor groove binders. Moreover, these conjugates exhibit favorable cytotoxicity against L1210 and HeLa cell lines, much improved in comparison to cis-platin and lower cytotoxicity toward BALB. This research paves a new direction towards the discovery of antitumor drugs. Recently, Rotaru et al. has developed a new fluorescent anthracene-based pyridyl-indolizine derivative (conjugate **V**) via “click” chemistry at the first position of the indolizine core to test their DNA binding efficacy and potential application towards anticancer treatment [187]. Agarose gel electrophoresis, UV–vis and fluorescence experiments along with molecular docking simulations has revealed that conjugate **108** (Figure 17) exhibits higher affinity for the DNA than its precursor containing only a pyridyl-indolizinic skeleton (conjugate **107**) owing to much lower values of binding energy and dissociation constant of the corresponding U-DNA complex.

### 4. Major groove binders

In general, biological macromolecules such as proteins interact with the major groove of ds-DNA via hydrogen bond interactions. In 2012, a detailed review on natural products DNA major groove binders such as pluramycins, aflatoxins, azinomycins, leinamycins, aminosugars, neocarzinostatins was reported, including their binding mechanisms and sequence specificity [188]. The authors clearly demonstrated how major groove binding molecules could block access to various transcription factors by binding to a specific DNA sequence. These natural products primarily act as intercalators; however, some of them interact covalently via alkylation of the nucleophilic sites on DNA. In this section, we will focus on more recent advances in the emergence of modified aminoglycosides (AGs) as reversible major groove binders. AGs are electrostatically attracted to the phosphodiester backbone of nucleic acids due to their polycationic nature. Moreover, they can adapt various conformations due to their flexible ring composition in order to bind within different DNA groove widths. However, B-form duplex DNA has a much larger major groove and the non-aromatic nature of aminoglycosides limits their binding to the DNA major groove due to the lack of shape-complementarity. In this regard, chemical modifications on AGs will lead to the design of novel DNA binding ligands with improved sequence specificity.

**Figure 18:**
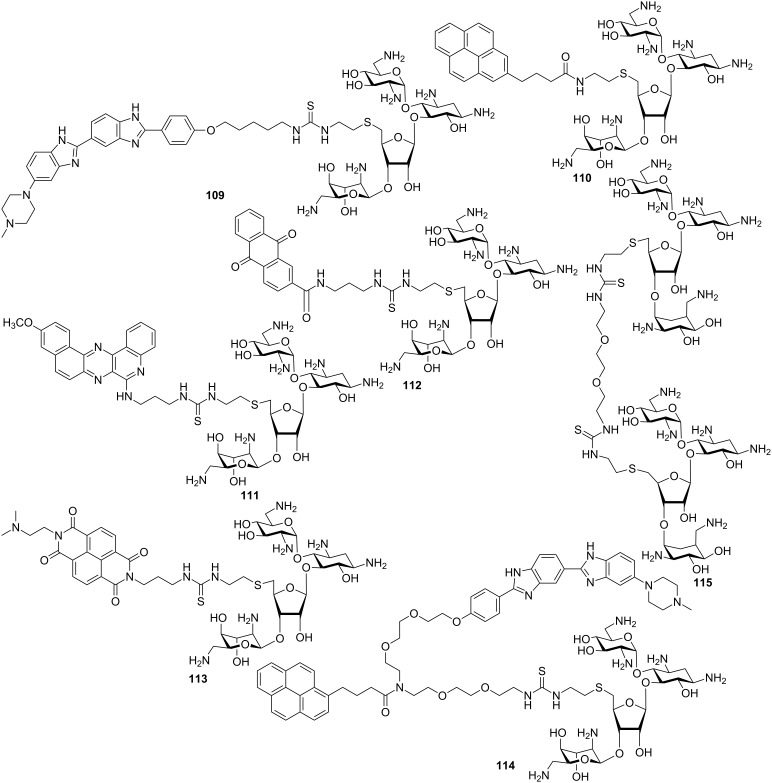
Aminoglycoside (neomycin)–Hoechst 33258/intercalator conjugates.

It has been observed that neomycin exhibits a much better shape complementarity with A-form DNA due to its narrower groove in comparison to B-DNA. Arya et al. investigated if neomycin, an effective A-form groove binder, could be inserted into the major groove of B-DNA by tethering neomycin with the well-known duplex selective groove binder Hoechst 33258. A neomycin–Hoechst 33258 conjugate **109** showed significant stabilization of DNA duplexes and destabilization of the DNA triplex which in turn, suggested that neomycin could be forced into the major groove of a B-form DNA duplex (Figure 18) [189].

Inspired by the earlier research, the triple recognition of B-DNA by a novel neomycin–Hoechst 33258–pyrene conjugate **114** was investigated in order to probe the molecular forces that dictate binding within the DNA grooves and base pairs by using spectroscopic, calorimetric, and viscometric techniques [12]. Conjugate **114** was found to bind stronger to B-DNA in comparison to all three constituents such as neomycin, pyrene and Hoechst 33258, thereby stabilizing DNA more efficiently. In addition, fluorescence titrations confirmed that the conjugate **114** could specifically recognize a contiguous stretch of nine A·T base pairs. The conjugate **114** was hypothesized to simultaneously recognize DNA via all three recognition motifs: major groove, minor groove, and intercalation and this research indeed paves the way for the development of multivalent DNA binding molecules. Kumar et al. reported a dimeric neomycin–neomycin conjugate **115** (Figure 18) with a flexible linker 2,20-(ethylenedioxy)bis(ethylamine) which could selectively bind to A·T-rich DNA duplexes preferentially over G·C-rich sequences confirmed via ITC, CD, FID, and UV thermal denaturation experiments [190]. Moreover, dimeric conjugate **115** exhibits higher affinity for the major groove of A-tract sequences over those containing alternating A·T bases. In addition, conjugate **115** destabilizes poly(dA)·2poly(dT) triplex but stabilizes poly(dA)·poly(dT) duplex, as opposed to neomycin monomer, suggesting the major groove as the binding site.

#### 4.1. Shape and nucleic acid selectivity (DNA vs. RNA)

One of the major concerns in nucleic acid recognition will be achieving selectivity: selectivity of one form of DNA over the other forms/sequences/shapes and DNA versus RNA selectivity. Previous work has shown that using designed molecules, both types of selectivity can be achieved. Using a competition dialysis assay, it was shown that neomycin is an A-form selective ligand over B-form structures irrespective of its constituent type (DNA or RNA) [191–192]. In a striking contrast, thiourea linked dimeric neomycin conjugates exhibited complete reversal of target selectivity from A-form triplex DNA to B-form duplex DNA structures [193]. Further investigations using a series of thiourea linked neomycin dimers spaced by different linker sizes revealed high affinity (*K*_a_ = 2.26 × 10^8^ M^−1^) binding for B-DNA over other forms of DNA. A FID based assay involving 512 DNA duplexes of different sequence compositions revealed that neomycin dimers prefer to bind DNA duplex with the AT-tract [190]. The neomycin dimer **115** (Figure 18) binds to short oligonucleotides (12 mer) with 1:1 ligand to DNA duplex stoichiometry and show a binding site size of 11–12 base pairs with the polymeric DNA. A complete thermodynamic study of neomycin dimer **115** binding to a B-DNA sequence revealed that the first binding event (the high affinity site) is entropically driven and that the ionic strength dependence of the binding is highly dependent on the electrolytic contribution [194]. The neomycin dimers also displayed length dependent shape recognition of the B-DNA [195].

**Figure 19:**
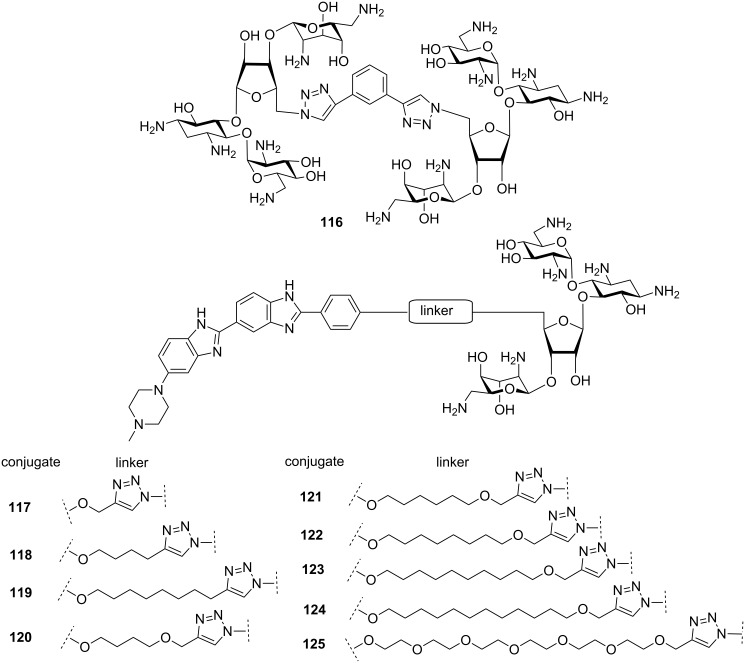
Chemical structures of triazole linked neomycin dimers and neomycin–bisbenzimidazole conjugates.

Dimerization of neomycin units using more rigid linkers (triazole linkers) than the thiourea linkers resulted in enhanced binding and more selective recognition of a TAR-RNA conformation over the DNA duplex structure [196–197]. The triazole linked neomycin dimer **116** (Figure 19) displayed close to nanomolar affinity (*K*_a_ = 1.39 × 10^8^ M^−1^) and 1:1 binding stoichiometry with a biologically relevant truncated model RNA sequence of TAR. In this case also, the binding was found to be dependent on the linker length joining the two neomycin units and the neomycin dimer conjugates thermally stabilized the TAR RNA structure by up to 10 °C. The neomycin dimers exhibited much improved cytopathic effects in MT-2 cells than neomycin alone [196]. These results showed that subtle changes in the linker composition bring profound differences in the DNA versus RNA nucleic acid selectivity. The linker length was found to have a significant and profound effect in the DNA versus RNA selectivity of a series of neomycin–bisbenzimidazole conjugates. It was found that neomycin–bisbenzimidazole conjugates **117**–**125** with short linkages (up to 11 atoms) stabilized a 12mer duplex DNA d(CGCAAATTTGCG)_2_ better than its RNA equivalent r(CGCAAAUUUGCG)_2_. However, neomycin–bisbenzimidazole conjugates with long linkers (15 atoms or higher) stabilized the RNA duplex sequence r(CGCAAAUUUGCG)_2_ better than the DNA sequence d(CGCAAATTTGCG)_2_ [198]. The unique selectivity of neomycin–bisbenzimidazole conjugates with long linkers towards RNA duplex was attributed to a linker dependent intercalation of the bisbenzimidazole unit into the RNA duplex, which was maximum (74 °C) with the longest linker (23 atoms). The dual binding of the conjugates allows both neomycin and bisbenzimidazole units binding in a complementary way to impart thermal stabilization of the RNA duplex [198]. The bisbenzimidazole units of the neomycin–bisbenzimidazole conjugates were earlier reported to bind in the minor groove of the DNA [199].

### 5. Alkylators

Covalent interaction between small molecules and DNA is usually irreversible, which leads to inhibition of DNA functions such as transcription or replication resulting in subsequent cell death. The small molecules can change the overall conformation by cross-linking to the DNA duplexes. However, their low selectivity reflects in their high toxicity in normal cells. Thus, in order to solve this issue, several researchers have designed and developed various synthetic analogs of existing drugs having much improved sequence specific DNA selectivity with reduced side effects, which are discussed in the following sections.

Alkylating agents are strong electrophilic compounds that react chemically with nucleophilic moieties of DNA or proteins to form covalent bonds by transferring an alkyl group to DNA. Their cytotoxicity results from the alkylation of DNA bases that can irreversibly inhibit essential DNA processes such as DNA replication and/or transcription. Nitrogen mustards, derived from sulfur mustards, including bendamustine, estramustine, melphalan, chlormethin, chlorambucil, were the first alkylating agents used for the treatment of leukemias and lymphomas. Alkylation occurs via the formation of an aziridinium ion followed by nucleophilic attack by the N7 of guanine [200]. The other well-known alkylators include platinum derivatives (cis-platin, carboplatin, oxaliplatine), oxazaphosphorines (cyclophosphamide, ifosfamide, trofosfamide), ethylene imines (mitomycin C, thiotepa, altretamine), nitrosoureas (MNU, MNNG, BCNU, CCNU, nimustine), triazenes and hydrazines (dacarbazine temozolomide Procarbazine) [201], trabectidine and so on [21].

**Figure 20:**
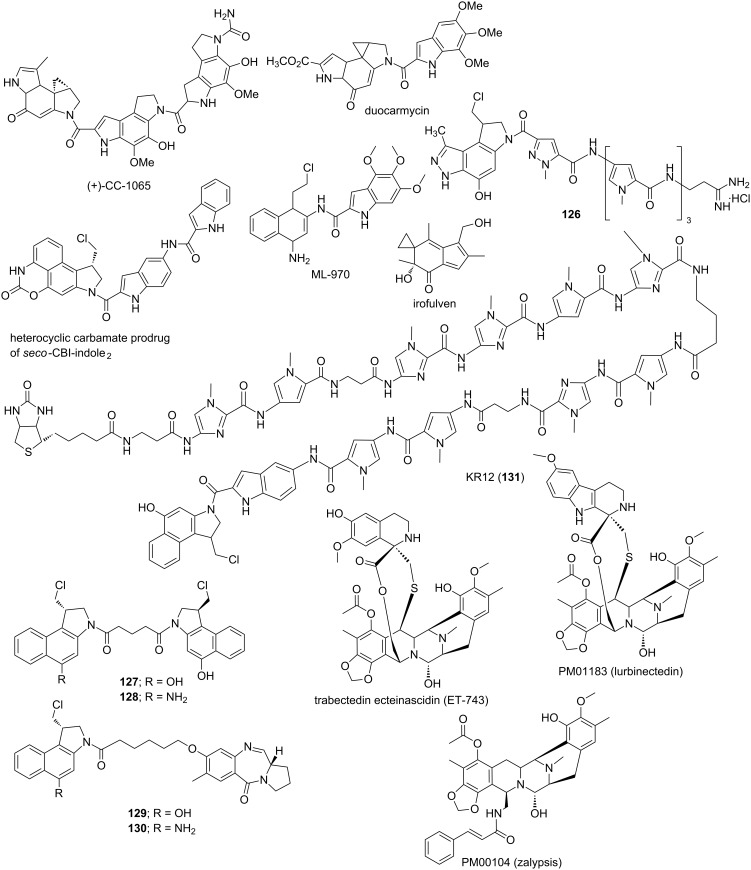
Representative examples of naturally occurring and synthetic analogs of DNA binding alkylating agents.

In the last few decades, a plethora of natural products and their synthetic analogs were tested for their antineoplastic effect which includes (+)-CC-1065, duocarmycin SA, irofulven, ML-970, *seco*-CBI-indole_2_ and so on (Figure 20). (+)-CC-1065 and duocarmycin SA are known antitumor drugs, isolated from *Streptomyces species*, which primarily act as minor groove alkylators by forming adenine N3 adducts in A·T-rich regions via the electrophilic cyclopropylindol (CPI) subunit [202–203]. However, these natural products showed significantly reduced antitumor activity mostly due to their low water solubility. Baraldi et al. reported a series of hybrid conjugates by tethering polypyrrole minor groove binders, derived from distamycin A and two pyrazole analogues of the CPI unit of the potent antitumor antibiotic (+)-CC-1065 in order to enhance potency, specificity and water solubility of pyrazole CPI analogs [204]. Conjugate **126** (Figure 20) was found to be extremely cytotoxic with IC_50_ values for the different tumor cell lines ranging from 7 to 71 nM.

Additionally, it exhibited the strongest DNA alkylation activity via sequence-specific alkylation of the third adenine located in the sequence 5′-ACAAAAATCG-3′ [204]. The high activity of tripyrrole conjugate **126** than mono- and dipyrrole analogs might result from its stronger binding with in the minor grooves due to multiple hydrogen bonding and van der Waals forces. However, higher toxicity of these natural products and their synthetic analogs forced the researchers to develop less toxic analogs. The newly identified indole-carboxamide ML-970 represents another synthetic derivative which binds the A·T-rich DNA minor groove and alkylates DNA. In addition, it shows potent cytotoxic activity, with an average GI_50_ of 34 nM with much lower myelotoxicity in comparison to (+)-CC-1065 and duocarmycins [205]. Another heterocyclic carbamate prodrug of *seco*-CBI-indole_2_ was reported which represents a new member of a class of hydrolyzable prodrugs of the duocarmycin and CC-1065 family of natural products [206]. This prodrug is activated by the hydrolysis of the carbamate residue, thereby slowly releasing the active free form of the drug with no residual byproduct (CO_2_). Thus, its slow free drug release allows the safe and efficacious administration of much higher doses than the parent-alkylating agent. Tercel et al. recently developed two new sets of DNA monoalkylating agents **127**, **128** (CBI–CBI dimer) and **129**, **130** [CBI–pyrrolobenzodiazepine (PBD) dimer], with phenol-CBI and amino-CBI residues and their cytotoxicity against nine human tumor cell lines were tested [207]. Interestingly, **128** and **130**, amino-CBI analogs found to be less cytotoxic (2- to 190-fold reduction in potency depending on the particular compound and cell line) in comparison to their phenol analogs **127** and **129** (Figure 20). Irofulven, a semisynthetic derivative of the mushroom-derived compound illudin S, is another extremely promising antitumor agent for solid tumor cells. Its mechanism of action involves an activation step in which nucleophilic attack on the α,β-unsaturated ketone by thiol or NADPH leads to opening of the cyclopropane ring, which results in alkylation of protein and DNA [208].

Recently, Lin et al. reported another attractive versatile sequence-specific DNA alkylating agent (KR12, **131**) by tethering well-known minor groove binder Py–Im polyamides with an alkylating agent such as *seco*-CBI (Figure 20) [209]. The authors have identified KR12 binding sites in the human LS180 colorectal cancer genome and the reduction of KR12-bound gene expressions was also observed. Another marine alkaloid trabectedin (ET-743) comprising of three fused tetrahydroisoquinoline rings has been introduced into clinical trial for the treatment of soft tissue sarcoma. Two of these sulfide-linked substituted isoquinoline rings take part in minor groove binding through covalent interaction with the third ring protruding from DNA duplex allowing interactions with adjacent nuclear proteins [210]. ET-743 interferes with several transcription factors and DNA binding proteins via preventing protein binding by distorting DNA structure. Two other synthetic tetrahydroisoquinolone alkaloid derivatives have been developed. PM01183 (lurbinectedin) [211] and PM00104 (Zalypsis^®^) [212], which showed broad range of chemotherapeutic activity against solid human tumor cell lines are currently in phase II trials. They both act as DNA binding agents, thereby causing inhibition of the cell cycle and transcription. Varadarajan et al. has developed a strategy for overcoming the deficiencies in current DNA-alkylating chemotherapy drugs by designing a site-specific DNA-methylating agent that can target cancer cells because of its selective uptake via glucose transporters, which are overexpressed in most cancers. A glucosamine unit, which can facilitate uptake via glucose transporters, was conjugated to one end of a bispyrrole triamide unit, which is known to bind to the minor groove of DNA at A/T-rich regions and led to increased activity against resistant glioblastoma cells [213].

### 6. Pyrrolobenzodiazepines (PBDs)

**Figure 21:**
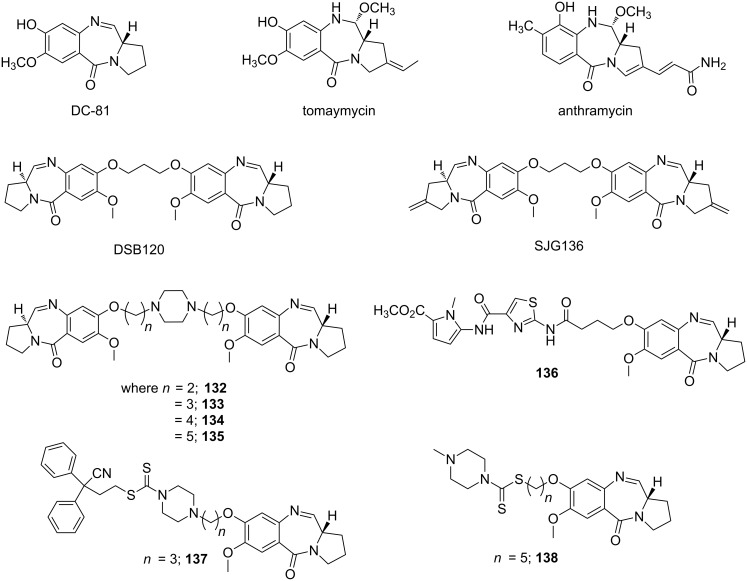
Chemical structures of naturally occurring and synthetic analogs of pyrrolobenzodiazepines.

Pyrrolobenzodiazepines (PBDs) are a class of naturally occurring sequence-selective DNA alkylating agents with antitumor properties, which include DC-81, tomaymycin, and anthramycin, isolated from various actinomycetes (Figure 21). The antitumor activity of these classes of molecules results from the sequence selective covalent binding with the 2-amino group of guanine bases in the minor groove of duplex DNA to the electrophilic imine of the diazepine ring. Anthramycin, isolated in the 1950s, is an active antitumor agent and exhibits antineoplastic activity against various types of tumors including Ehrlich solid carcinoma, sarcoma, epidermal carcinoma and leukemia L1210 cells [214].

However, its high cardio toxicity limits clinical application. In order to enhance their DNA binding affinity, several C8-diether-linked DC-81 dimers such as DSB-120 (dimer of DC-81) were synthesized [215–216]. Unfortunately, these dimers did not exhibit expected in vivo antitumor activity probably due to the low bioavailability and excessive electrophilicity at the N10–C11 imine moiety [217]. This led to develop another PBD dimer (SJG-136) linked by a propane-1,3-diether, which exhibited significant in vivo potential for leukemia treatment. SJG-136 has recently passed phase II clinical trials in patients with leukemia and ovarian cancer. Kamal et al. designed a series of novel PBD dimers **132**–**135** comprising of two DC-81 subunits tethered via piperazine side-armed-alkane spacer [217]. These conjugates, especially conjugate **134**, exhibit much improved cytotoxicity than DSB-120 in nine different human cancer cell lines. The author’s demonstrated installation of a piperazine ring in the middle of such an alkanedioxy linker results in several hydrophobic interactions, which in turn, enhances DNA binding ability, confirmed via DNA thermal denaturation studies. A set of novel hybrid conjugates by tethering PBD with polyamides, well-known DNA minor groove binders, was designed by Thurston et al. in order to explore structure/sequence selectivity relationships and target gene promoter regions [218]. Conjugate **136** comprising of *N*-methylpyrrole and a thiazole residue exhibits greater DNA binding affinity as well as selectivity for inverted CCAAT sequences within the topoisomerase IIα promoter region. Recently, Kamal et al. reported a set of C8-linked dithiocarbamate/piperazine bridged PBD conjugates and their cytotoxic potential and DNA binding ability were evaluated [219]. Conjugate **137** has shown promising cytotoxicity against 33 cell lines in nine cancer phenotypes with GI_50_ values of <0.99 μM. Thermal denaturation (Δ*T*_m_) studies revealed that by the introduction of *N*-methylpiperazine dithiocarbamate with five-membered alkane spacer to the PBD core increased the DNA-binding activity considerably in conjugate **138** (Δ*T*_m_ = 10.9 °C, Figure 21). Thurston et al. recently reported a thorough review on the topic, covering the recent developments, SARs and biological applications of PBDs [16].

## Conclusion

Regulation of DNA functions with the interference of small molecule DNA binding agents is an established and ongoing area of nucleic acid targeted drug discovery. The clinical success, coupled with high cytotoxicity of DNA binding anticancer agents such as doxorubicin and cis-platin over the past four decades challenges us to design novel agents with reduced toxicity and alternative mechanisms. As covered in this review, new DNA binders are rapidly gaining a foothold in somewhat less explored domains of their application as antibacterial, antifungal and antiparasitic agents beyond their repertoire as anticancer agents. Many of the known sequence specific polyamides have been successfully developed as hairpins, H-pins and hybrid conjugates for enhanced recognition of contiguous DNA bases. The molecules covered in this review show that they indeed are capable of disrupting DNA-transcription factor interactions with high affinity highlighting their emerging importance in chemical biology and potential therapeutics. Recent reports have also shown that end modification of classical bisbenzimidazole (such as Hoechst 33258) based minor groove binding agents leads to dramatic changes in DNA binding, selectivity in bacterial versus human topoisomerase, cellular internalization and cytotoxicity [123]. These findings highlight the sensitivity of DNA sequence selective binders to even modest changes in the chemical structure of the target ligand.

An important aspect of hybrid drug design is the role of linker length and composition on target selectivity and affinity. Optimization of the linker length is an important aspect of fragment-based drug design and appropriate linkage assessment is crucial in optimizing the target binding and cellular uptake of nucleic acid binding ligands. The discoveries summarized in this report reflect the enormous potential, challenges and expanding diversity of DNA targeted drugs in addressing current therapeutic challenges.
